# Supplementary material for: Identification of five sleep-biopsychosocial profiles with specific neural signatures linking sleep variability with health, cognition, and lifestyle factors
Source: PLoS Biol. 2025 Oct 7;23(10):e3003399. doi: 10.1371/journal.pbio.3003399 (PMC12503254; doi:10.1371/journal.pbio.3003399)
Supplement: S1 Text — Table A. Sleep and nonsleep-biopsychosocial measures used in the CCA (indicated in black), or in post hoc analyses (indicated in blue), or in both (indicated in green). Table B. CCA loadings and z-scores for sleep and biopsychosocial measures for LCs 1–5. Loadings with significant bootstrapped z-scores that survived FDR correction (q < 0.05) are indicated in bold. Table C. Cross-validated canonical correlation analysis. Significant p-values are indicated in bold. Table D. Post hoc associations between sleep (or biopsychosocial; BPS) composite scores and sociodemographic measures, as well as physical and mental health measures. Associations using continuous measures (e.g., age, education years) were tested with Pearson correlations, while categorical measures (e.g., sex, race) were assessed with t-tests or analyses of variance. Associations indicated in bold were found to be statistically significant after FDR correction (q < 0.05). Tests with <5 subjects in a category were not computed because of too little variance. Table E. Correlations between sleep (or biopsychosocial) loadings in control analyses with those from the original analysis. Fig A. Latent Components LC6 and LC7. (A) Sleep loadings (left) and top 15 strongest biopsychosocial (BPS) loadings (right) for LC6. (B) Sleep loadings (left) and top 15 strongest biopsychosocial (BPS) loadings (right) for LC7. Positive values on sleep (blue) loadings indicate worse outcomes while positive values on biopsychosocial loadings reflect higher magnitude on these measures. Error bars indicate bootstrapped-estimated confidence intervals (i.e., standard deviation) and measures in bold indicate statistical significance. Fig B. Post hoc associations between CCA mean composite scores and RSFC, highlighting beta coefficients between subcortical regions and cortical networks. Fig C. Control analyses for post hoc associations with RSFC. (Left panel) GLM analysis using RSFC data that underwent CompCor instead of global signal re [file pbio.3003399.s002.pdf]

Supporting Information

Full Title:

Identification of five sleep-biopsychosocial profiles with specific neural signatures linking sleep variability with health, cognition and lifestyle factors

Short Title: Sleep and biopsychosocial profiles

Authors:

Aurore A. Perrault[1,2,3Δ*], Valeria Kebets[4,5,6,7,8Δ*], Nicole M. Y. Kuek[4,5,6], Nathan E. Cross[1,2,9], Rackeb Tesfaye[8], Florence B. Pomares[1,2], Jingwei Li[4,5,6,10,11], Michael W.L. Chee[5], Thien Thanh Dang-Vu[1,2], B.T. Thomas Yeo[4,5,6,12,13,14]

Affiliations:

1. Sleep, Cognition and Neuroimaging Lab, Department of Health, Kinesiology and Applied Physiology & Center for Studies in Behavioral Neurobiology, Concordia University, Montreal, QC, Canada
2. Centre de Recherche de l'Institut Universitaire de Gériatrie de Montréal, CIUSSS Centre-Sud-de-l'Ile-de-Montréal, QC, Canada
3. Sleep & Circadian Research Group, Woolcock Institute of Medical Research, Macquarie University, Sydney, NSW, Australia
4. Department of Electrical and Computer Engineering, National University of Singapore, Singapore
5. Centre for Sleep and Cognition & Centre for Translational Magnetic Resonance Research, Yong Loo Lin School of Medicine, National University of Singapore, Singapore
6. N.1 Institute for Health, National University of Singapore, Singapore
7. McConnell Brain Imaging Centre (BIC), Montreal Neurological Institute (MNI), McGill University, Montreal, QC, Canada
8. McGill University, Montreal, QC, Canada
9. School of Psychology, University of Sydney, NSW, Australia
10. Institute of Neuroscience and Medicine (INM-7: Brain and Behavior), Research Center Jülich, Germany
11. Institute of Systems Neuroscience, Medical Faculty and University Hospital Düsseldorf, Heinrich Heine University Düsseldorf, Germany
12. Department of Medicine, Human Potential Translational Research Programme & Institute for Digital Medicine (WisDM), Yong Loo Lin School of Medicine, National University of Singapore, Singapore
13. Integrative Sciences and Engineering Programme (ISEP), National University of Singapore, Singapore
14. Martinos Center for Biomedical Imaging, Massachussetts General Hospital, Charlestown, MA, USA

Δ Authors contributed equally.

*Corresponding authors:
Aurore A. Perrault, Valeria Kebets
aurore.perrault@gmail.com ; valkebets@gmail.com

Supplementary Results A

Latent Components 6 and 7 (LCs 6-7)

Compared to LCs 1-5, LC6 and LC7 were considered less interpretable and relevant with regards to established sleep literature. In addition, the lack of either a single driven sleep variable (like LC2-5) or common directionality in sleep patterns complicated the interpretation of the profiles. Nonetheless, the sleep-biopsychosocial covariance patterns are described below.

LC6 was characterised by longer sleep latency without sleep disturbance or sleep dissatisfaction. In terms of biopsychosocial variables, LC6 was driven by withdrawn and avoidant personality problems, short reaction time on a gambling task, low blood pressure, not being in a relationship and the use of alcohol (**Figure A in S1 Text)**.

Meanwhile, LC7 was driven by low sleep efficiency (i.e., long time spent in bed without sleeping) and short sleep duration as well as short reaction time on working memory and emotion processing cognitive tasks (**Figure A in S1 Text**).

Supplementary Results B

Post-hoc associations with socio-demographics, health, and family history of mental health

We found several significant associations between LC composite scores and biopsychosocial factors (**Table D in S1 Text**).

In LC1, we found a significantly negative correlation between higher sleep and biopsychosocial composite scores and education level, and significant differences in sleep and biopsychosocial composite scores between household income categories (i.e., generally higher scores in those living in a low-income household). Biopsychosocial composite scores were also higher in Hispanic/Latino participants and in participants with a paternal history of depression.

In LC2, we found a significantly positive correlation between higher sleep and biopsychosocial composite scores and education level; significantly higher biopsychosocial composite scores in participants from lower household income); lower composite sleep scores in participants working full-time vs. part-time vs. those not working; higher sleep scores in those not in school; higher sleep composite scores in participants with a paternal history of depression, higher biopsychosocial composite scores in those with a maternal history of anxiety or a paternal history of addiction.

In LC3, we found significant differences in sleep composite scores between participants from different racial backgrounds (i.e., higher scores in participants who identified as White), and a significant negative correlation between sleep composite scores and BMI.

In LC4, we found a significant association between higher sleep and biopsychosocial composite scores and higher age as well as lower education level, and between higher sleep scores and higher BMI. There were also significant differences in sleep composite scores between participants from different racial backgrounds. We also found higher biopsychosocial composite scores in participants working full time but also in those not in school. Finally, we find lower biopsychosocial scores in participants with a paternal history of anxiety.

Finally in LC5, we found higher sleep and biopsychosocial composite scores in female participants; a significant association between higher sleep and biopsychosocial composite scores and lower education level; and significant differences between participants from different racial backgrounds.

Supplementary Results C

Control per biological sex analyses

Additionally, we ran two separate CCA in respectively only female and only male participants.

In the model with female participants only (N=414), all 7 LCs were significant (all ps=0.0002 except LC7 p=0.0021). The first two LCs are highly similar to the first two LCs from the original model, while the rest of the LCs show low to moderate correlation to the original LCs (**Table E in S1 Text**). In the CCA with male participants only (N=356), all LCs except LC7 were found to be significant (all ps < 0.001 except LC7 p=0.06). Again, the first two LCs are highly similar, while the rest of the LCs is low to moderately similar to the original LCs (**Table E in S1 Text)**. The sex specificity highlights the meaningful interplay between biological sex and individual differences in sleep and biopsychosocial factors, however this could also be due to the reduced sample size in each sex-specific model.

Control RSFC analyses

We computed some control GLM analyses with RSFC data (**Figure C in S1 Text**).
First, we computed a GLM using RSFC data that underwent CompCor instead of global signal regression, which showed high correlations with the main GLM analysis (LC1: r=0.75; LC2: r=0.76; LC3: r=0.78; LC4: r=0.51; LC5: r=0.77).

Next, we computed a GLM analysis after excluding subjects that likely fell asleep in the scanner (N=100), which showed high correlations with the main GLM analysis (LC1: r=0.90; LC2: r=0.87; LC3: r=0.95; LC4: r=0.95; LC5: r=0.95).

Third, we computed a GLM analysis between RSFC and *sleep* composite scores (instead of averaged composite scores), which showed moderate to high correlations with the main GLM analysis (LC1: r=0.72; LC2: r=0.60; LC3: r=0.74; LC4: r=0.52; LC5: r=0.66).

We did the same between RSFC and *biopsychosocial* composite scores (instead of averaged composite scores), and found moderate to high correlations with the main GLM analysis (LC1: r=0.69; LC2: r=0.62; LC3: r=0.63; LC4: r=0.46; LC5: r=0.67).

Finally, we computed integration and segregation measures on the average RSFC matrix of the whole sample to compare our findings per LC with the average (**Figure E in S1 Text**).

Control analyses with asleep participants

We computed *post-hoc* t-tests to assess differences in sleep (or biopsychosocial) composite scores between participants who likely stayed awake (N=623) in the scanner vs. those who likely fell asleep in the scanner (N=100). Participants that likely fell asleep in the scanner had significantly higher biopsychosocial composite scores on LC1 (t=2.93, p=0.003), significantly lower sleep composite scores on LC3 (t=-2.42, p=0.016), and significantly higher sleep (t=2.25, p=0.025) and biopsychosocial composite scores on LC4 (t=2.85, p=0.005) compared to those that likely stayed awake in the scanner (**Figure D in S1 Text).**

When inspecting the distribution of sleep and biopsychosocial composite scores of these participants, they did not appear to be driving any of the LCs (**Figure F in S1 Text**).

Supplementary Tables

Table A

Sleep and non-sleep biopsychosocial measures used in the CCA (indicated in black), or in post-hoc analyses (indicated in blue), or in both (indicated in green).
References of the different measures used can be found in the primary HCP paper *(Van Essen et al., NeuroImage, 2013 – Reference 38 in main manuscript)*

Domain	Scale	Subdomain	Measure
Sleep	Pittsburgh Sleep Quality Index	Component 1: Sleep satisfaction	
		Component 2: Sleep latency	
		Component 3: Sleep duration	
		Component 4: Sleep efficiency	
		Component 5: Sleep disturbance	
		Component 6: Sleep meds	
		Component 7: Daytime functioning	
Socio-demographics			Age
			Sex
			Education level (years)
			Race
			Ethnicity
			Employment status
			Household income
			School status
			Relationship status
Physical health		Physical function	Body mass index (BMI)
			Blood pressure*
			Hematocrit levels**
Cognition (outside the scanner)	NIH Toolbox (https://www.nihtoolbox.org/)	Episodic memory	Picture sequence memory test
		Executive function, cognitive flexibility	Dimensional change card sort test
		Executive function, inhibition	Flanker inhibitory control and attention test
		Language, reading decoding	Oral reading recognition test

		Language, vocabulary comprehension	Picture vocabulary test
		Processing speed	Pattern comparison processing speed test
		Working memory	List sorting working memory test
	Penn Progressive matrices	Fluid intelligence	Accuracy
			RT
	Variable Short Penn Line Orientation Test	Visual-spatial processing	Accuracy
			RT
	Short Penn Continuous Performance Test (Number/Letter Version)	Sustained attention	True positives
			Sensitivity
			Specificity
	Penn Word Memory Test, Form A	Verbal episodic memory	Accuracy
			RT
	Penn Emotion Recognition Test	Emotion processing	Accuracy- Anger
			Accuracy- Fear
			Accuracy- Happy accuracy
			Accuracy- Neutral accuracy
			Accuracy- Sad accuracy
			Accuracy- Total
			RT- Total
	Delay discounting task	Self-regulation, impulsivity	Area under the curve (AUC) for $200
			Area under the curve (AUC) for $40,000
Cognition (in-scanner)	Language task	Language processing	Accuracy
			RT
	Relational task	Relational processing	Accuracy
			RT
	Social task	Social cognition, theory of mind	Accuracy random stimuli rated random
			RT random stimuli rated random
			Accuracy social stimuli rated social
			RT social stimuli rated social
	Working memory n-back task	Working memory, cognitive control	Accuracy
			RT
	Gambling task	Incentive processing	Accuracy smaller prediction
			RT smaller prediction
			Accuracy larger prediction
			RT larger prediction
	Hariri Emotion task	Emotional processing	Accuracy
			RT
Affect	NIH Toolbox (https://www.nihtoolbox.org/)	Negative affect	Anger- affect
			Anger- hostility
			Anger- physical aggression
			Fear- affect
			Fear- somatic arousal
			Sadness
		Psychological well-being	Positive affect
			General life satisfaction
			Meaning and purpose
		Social relationships	Emotional support
			Instrumental support

			Friendship
			Loneliness
			Perceived hostility
			Perceived rejection
		Stress and self-efficacy	Perceived stress
			Self-efficacy
Sensory	NIH Toolbox (https://www.nihtoolbox.org/)	Sensory processing	Pain interference
Personality	Costa and McRae Neuroticism/Extroversion/Openness Five Factor Inventory (NEO-FFI)	Personality traits	Agreeableness
			Openness to experience
			Conscientiousness
			Neuroticism
			Extroversion/introversion
Mental health	Achenbach Adult Self-Report (ASR)	Life function, psychiatric clinical symptoms	Anxious depressed
			Withdrawn
			Somatic complaints
			Thought problems
			Rule breaking behavior
			Aggressive behavior
			Attention problems
			Intrusive problems
			Other problems
			Internalizing problems
			Externalizing problems
			Total problems
			Critical items
			DSM Anxiety problems
			DSM ADHD problems
			DSM Hyperactivity problems
			DSM Depressive problems
			DSM Avoidant personality problems
			DSM Antisocial personality problems
			DSM Somatic problems
			DSM Inattention problems
	Semi-Structured Assessment for the Genetics of Alcoholism (SSAGA)	Psychiatric clinical symptoms	Agoraphobia
			Childhood conduct
			Major depressive episodes
			Number of depressive symptoms
			Panic disorder
		Family history of psychiatric and neurological disorders	Maternal history of schizophrenia or psychosis
			Paternal history of schizophrenia or psychosis
			Maternal history of depression
			Paternal history of depression
			Maternal history of bipolar disorder
			Paternal history of bipolar disorder
			Maternal history of anxiety
			Paternal history of anxiety
			Maternal history of drug or alcohol problems
			Paternal history of drug or alcohol problems

			Maternal history of Alzheimer's disease or dementia
			Paternal history of Alzheimer's disease or dementia
			Maternal history of Parkinson's disease
			Paternal history of Parkinson's disease
			Maternal history of Tourette's syndrome
			Paternal history of Tourette's syndrome
Substance use	Semi-Structured Assessment for the Genetics of Alcoholism (SSAGA)	Substance use, abuse and dependence	Alcohol age first use
			Drinks per day past 12 months
			Frequency alcohol use past 12 months
			Frequency drunk past 12 months
			Drinks per day heaviest period
			Frequency alcohol use heaviest period
			Frequency drunk heaviest period
			Lifetime alcohol abuse diagnosis
			Lifetime alcohol abuse symptoms
			Lifetime alcohol dependence diagnosis
			Lifetime alcohol dependence symptoms
			Times used cocaine
			Times used hallucinogens
			Times used marijuana
			Times used nonmarijuana illicit drugs
			Times used opiates
			Times used sedatives
			Times used stimulants
			Marijuana dependence
	Fagerstrom Test for Nicotine Dependence	Nicotine dependence	History of smoking
			Currently smoking

**Blood pressure was categorized into (i) hypotension (systolic blood pressure (BP) $\leq$ 90 and/or diastolic BP $\leq$ 60), (ii) normal BP (systolic BP < 120 and diastolic BP = 80), (iii) elevated BP (systolic BP between 120-129 and/or diastolic BP < 80), (iv) hypertension stage 1 (systolic BP between 130-139 and/or diastolic BP between 80-89), and (v) hypertension stage 2 (systolic BP > 140 and/or diastolic BP $\geq$ 90).*

*** Hematocrit levels were averaged across the day 1 and day 2 measurements.*

Table B

CCA loadings and z-scores for sleep and biopsychosocial measures for LCs 1-5. Loadings with significant bootstrapped z-scores that survived FDR correction ($q < 0.05$) are indicated in bold.

Note that LC1, LC2 and LC5 are inverted in our manuscript with regards to its interpretation, as the directionality of CCA loadings and scores is arbitrary.

Variable	LC1 loading (z-score)	LC2 loading (z-score)	LC3 loading (z-score)	LC4 loading (z-score)	LC5 loading (z-score)
Comp1 Sleep satisfaction (PSQI)	**-0.72 (-4.06)**	0.22 (1.19)	-0.23 (-1.14)	0.21 (0.91)	**0.44 (2.37)**
Comp2 Sleep latency (PSQI)	**-0.72 (-3.88)**	**0.35 (2.09)**	0.21 (0.97)	-0.16 (-0.75)	-0.06 (-0.30)
Comp3 Sleep duration (PSQI)	-0.22 (-1.00)	0.13 (0.51)	-0.20 (-0.72)	**0.83 (3.86)**	-0.14 (-0.43)
Comp4 Sleep efficiency (PSQI)	-0.33 (-1.32)	0.22 (1.25)	-0.18 (-0.83)	-0.15 (-0.54)	-0.18 (-0.58)
Comp5 Sleep disturbance (PSQI)	**-0.57 (-3.23)**	0.17 (0.90)	-0.16 (-0.75)	-0.07 (-0.26)	**-0.64 (-3.57)**
Comp6 Sleep meds (PSQI)	**-0.39 (-2.09)**	-0.17 (-0.55)	**0.85 (5.71)**	0.13 (0.44)	-0.06 (-0.26)
Comp7 Daytime functioning (PSQI)	**-0.57 (-3.21)**	**-0.74 (-4.57)**	-0.33 (-1.19)	0.02 (0.08)	-0.03 (-0.18)
Employment status	**0.10 (1.81)**	-0.02 (-0.30)	-0.03 (-0.37)	**0.14 (1.87)**	-0.01 (-0.10)
Household income	**0.13 (1.73)**	**0.14 (1.67)**	-0.04 (-0.42)	0.14 (1.41)	**0.23 (2.73)**
School status	0.00 (-0.05)	**-0.17 (-2.35)**	0.06 (0.59)	**0.16 (1.89)**	-0.01 (-0.14)
Relationship status	0.02 (0.42)	0.00 (0.03)	-0.11 (-1.41)	0.04 (0.47)	0.11 (1.25)
BMI	-0.07 (-0.91)	0.10 (1.04)	**-0.23 (-2.72)**	0.11 (1.07)	-0.15 (-1.56)
Hematocrit	**-0.09 (-1.76)**	0.06 (1.09)	-0.04 (-0.53)	-0.06 (-0.88)	0.07 (1.11)
Blood pressure	0.02 (0.31)	0.11 (1.27)	-0.09 (-0.89)	-0.13 (-1.20)	**-0.23 (-2.19)**
Anxious depressed (ASR)	**-0.47 (-3.69)**	**-0.32 (-3.79)**	-0.07 (-0.56)	0.04 (0.38)	-0.15 (-1.53)
Withdrawn (ASR)	**-0.30 (-3.00)**	**-0.21 (-2.30)**	-0.15 (-1.47)	0.02 (0.22)	-0.12 (-1.17)
Somatic complaints (ASR)	**-0.62 (-3.83)**	-0.11 (-1.24)	0.03 (0.32)	-0.01 (-0.11)	**-0.16 (-2.12)**
Thought problems (ASR)	**-0.38 (-3.27)**	**-0.31 (-3.05)**	-0.17 (-1.36)	0.11 (0.94)	**-0.20 (-2.01)**
Rule breaking behavior (ASR)	**-0.30 (-3.16)**	**-0.17 (-2.11)**	-0.12 (-1.29)	0.04 (0.40)	-0.09 (-1.13)
Aggressive behavior (ASR)	**-0.35 (-2.91)**	**-0.21 (-2.47)**	-0.05 (-0.47)	0.16 (1.54)	**-0.20 (-2.23)**
Attention problems (ASR)	**-0.41 (-3.65)**	**-0.45 (-4.17)**	-0.24 (-1.60)	-0.05 (-0.36)	-0.02 (-0.22)
Intrusive problems (ASR)	-0.10 (-1.62)	**-0.12 (-1.75)**	-0.10 (-1.33)	0.01 (0.17)	-0.05 (-0.63)
Other problems (ASR)	**-0.48 (-3.57)**	**-0.34 (-3.82)**	-0.09 (-0.68)	0.05 (0.40)	**-0.18 (-1.86)**
Internalizing problems (ASR)	**-0.56 (-3.76)**	**-0.28 (-3.14)**	-0.07 (-0.58)	0.03 (0.25)	**-0.17 (-1.83)**
Externalizing problems (ASR)	**-0.34 (-3.04)**	**-0.22 (-2.60)**	-0.11 (-1.04)	0.10 (1.02)	**-0.16 (-1.83)**
Total problems (ASR)	**-0.54 (-3.63)**	**-0.36 (-3.70)**	-0.14 (-1.03)	0.05 (0.43)	**-0.17 (-1.76)**
Critical items (ASR)	**-0.44 (-3.27)**	**-0.40 (-4.37)**	-0.06 (-0.41)	0.08 (0.64)	**-0.22 (-2.17)**
DSM Anxiety problems (ASR)	**-0.50 (-3.44)**	**-0.27 (-3.02)**	-0.08 (-0.65)	0.12 (1.10)	**-0.18 (-1.93)**
DSM ADHD problems (ASR)	**-0.40 (-3.51)**	**-0.37 (-4.54)**	-0.08 (-0.60)	0.03 (0.30)	-0.06 (-0.65)
DSM Hyperactivity problems (ASR)	**-0.39 (-3.34)**	**-0.21 (-2.63)**	0.09 (0.91)	0.05 (0.51)	-0.12 (-1.48)
DSM Depressive problems (ASR)	**-0.61 (-3.99)**	**-0.32 (-3.47)**	-0.10 (-0.82)	-0.05 (-0.45)	-0.05 (-0.58)
DSM Avoidant personality problems (ASR)	**-0.26 (-3.18)**	**-0.20 (-2.31)**	-0.15 (-1.45)	-0.03 (-0.24)	-0.07 (-0.68)
DSM Antisocial personality problems (ASR)	**-0.30 (-2.79)**	**-0.18 (-2.13)**	-0.14 (-1.51)	0.06 (0.59)	-0.12 (-1.35)
DSM Somatic problems (ASR)	**-0.42 (-3.45)**	**-0.17 (-2.08)**	0.06 (0.64)	-0.01 (-0.07)	**-0.19 (-2.35)**
DSM Inattention problems (ASR)	**-0.31 (-3.32)**	**-0.42 (-4.40)**	-0.20 (-1.42)	0.01 (0.09)	0.00 (0.03)
Childhood conduct (SSAGA)	**-0.23 (-3.07)**	-0.05 (-0.55)	**-0.20 (-2.93)**	0.00 (0.03)	-0.10 (-1.21)
Panic disorder (SSAGA)	**-0.14 (-2.23)**	-0.04 (-0.58)	-0.01 (-0.12)	-0.04 (-0.57)	0.02 (0.23)
Agoraphobia (SSAGA)	-0.08 (-1.57)	-0.11 (-1.28)	**-0.20 (-2.78)**	-0.01 (-0.08)	0.02 (0.26)
Major depressive episodes (SSAGA)	**-0.33 (-3.51)**	**-0.23 (-3.09)**	0.03 (0.25)	-0.03 (-0.29)	-0.07 (-0.81)
Number of depressive symptoms (SSAGA)	**-0.35 (-3.49)**	**-0.23 (-3.22)**	0.01 (0.12)	-0.02 (-0.23)	-0.04 (-0.45)
Picture sequence memory accuracy (NIH)	0.00 (0.07)	0.04 (0.43)	**-0.22 (-2.81)**	**-0.16 (-1.74)**	0.02 (0.24)
Dimensional change card sort accuracy (NIH)	0.00 (-0.07)	0.09 (1.46)	0.01 (0.07)	0.01 (0.09)	0.07 (0.88)

Flanker inhibitory control attention accuracy (NIH)	0.02 (0.41)	0.10 (1.56)	-0.06 (-0.88)	0.07 (0.92)	0.03 (0.44)
Oral reading recognition accuracy (NIH)	-0.02 (-0.36)	**-0.20 (-2.70)**	0.03 (0.29)	-0.14 (-1.47)	**0.19 (2.13)**
Picture vocabulary accuracy (NIH)	-0.04 (-0.69)	**-0.13 (-1.97)**	0.00 (0.00)	0.03 (0.31)	**0.17 (2.35)**
Pattern comparison processing speed accuracy (NIH)	-0.01 (-0.24)	0.05 (0.71)	**-0.14 (-2.19)**	-0.05 (-0.57)	-0.06 (-0.68)
Penn Progressive matrices accuracy	0.01 (0.23)	**-0.20 (-2.58)**	-0.12 (-1.21)	**-0.17 (-2.02)**	0.07 (0.74)
Penn Progressive matrices RT	0.01 (0.09)	**-0.21 (-2.45)**	**-0.18 (-1.91)**	-0.12 (-1.28)	0.07 (0.69)
Variable short Penn line orientation accuracy	0.00 (-0.01)	-0.06 (-0.90)	-0.07 (-0.92)	**-0.14 (-1.78)**	0.11 (1.30)
Variable short Penn line orientation RT	-0.05 (-0.77)	-0.05 (-0.67)	**-0.17 (-2.43)**	-0.06 (-0.65)	0.02 (0.19)
Short Penn continuous performance true positives	**-0.09 (-1.83)**	-0.06 (-0.95)	0.01 (0.16)	-0.05 (-0.71)	0.04 (0.51)
Short Penn continuous performance sensitivity	-0.05 (-0.97)	0.01 (0.14)	0.07 (1.09)	-0.06 (-0.69)	0.15 (2.02)
Short Penn continuous performance specificity	0.04 (0.64)	-0.11 (-1.65)	-0.08 (-0.97)	-0.08 (-0.90)	0.08 (0.89)
Penn word memory test accuracy	-0.05 (-1.03)	0.07 (1.22)	-0.03 (-0.46)	-0.06 (-0.89)	-0.01 (-0.12)
Penn word memory test RT	-0.07 (-1.09)	0.00 (-0.01)	0.10 (1.31)	-0.10 (-1.17)	0.03 (0.39)
List sorting working memory accuracy	-0.03 (-0.54)	**0.16 (2.02)**	-0.14 (-1.53)	**-0.16 (-1.82)**	0.10 (1.02)
Language task accuracy	0.00 (-0.01)	-0.02 (-0.20)	-0.06 (-0.69)	**-0.19 (-2.27)**	**0.17 (1.90)**
Language task RT	**-0.12 (-1.84)**	-0.02 (-0.22)	0.03 (0.36)	**0.15 (1.83)**	-0.11 (-1.28)
Relational task accuracy	0.05 (0.70)	-0.05 (-0.54)	-0.01 (-0.10)	-0.16 (-1.62)	**0.25 (2.90)**
Relational task RT	0.06 (0.91)	-0.06 (-0.91)	-0.05 (-0.63)	0.00 (0.04)	0.01 (0.10)
Social task random stimuli rated random accuracy	0.02 (0.34)	**-0.13 (-1.84)**	0.05 (0.62)	**0.14 (1.75)**	-0.04 (-0.49)
Social task random stimuli rated random RT	0.01 (0.25)	0.12 (1.46)	-0.06 (-0.63)	**0.22 (2.52)**	0.04 (0.43)
Social task social stimuli rated social accuracy	**0.12 (2.11)**	0.01 (0.16)	-0.02 (-0.19)	**0.16 (2.27)**	0.07 (0.86)
Social task social stimuli rated social RT	0.01 (0.15)	**-0.13 (-1.71)**	-0.06 (-0.75)	**0.15 (1.79)**	0.01 (0.09)
Working memory task accuracy	0.10 (1.33)	-0.06 (-0.83)	-0.08 (-0.98)	-0.12 (-1.34)	**0.19 (2.39)**
Working memory task RT	**-0.12 (-2.06)**	0.04 (0.54)	0.05 (0.60)	0.01 (0.15)	-0.12 (-1.26)
Delay discounting task $200 AUC	0.13 (1.31)	**-0.18 (-2.30)**	-0.03 (-0.28)	**-0.22 (-2.18)**	0.03 (0.30)
Delay discounting task $40K AUC	0.07 (0.75)	**-0.16 (-1.90)**	-0.06 (-0.62)	-0.19 (-1.83)	0.09 (0.82)
Gambling task larger prediction accuracy	-0.07 (-1.24)	-0.08 (-1.30)	0.03 (0.37)	0.04 (0.51)	-0.06 (-0.84)
Gambling task smaller prediction accuracy	0.07 (1.24)	0.08 (1.30)	-0.03 (-0.37)	-0.04 (-0.51)	0.06 (0.84)
Gambling task larger prediction RT	-0.04 (-0.66)	-0.08 (-1.01)	0.13 (1.60)	0.04 (0.40)	-0.06 (-0.59)
Gambling task smaller prediction RT	-0.04 (-0.81)	-0.10 (-1.22)	0.11 (1.27)	0.07 (0.66)	0.00 (0.04)
Penn emotion recognition Anger accuracy	-0.06 (-1.01)	0.03 (0.56)	-0.02 (-0.26)	0.06 (0.87)	0.00 (-0.04)
Penn emotion recognition Fear accuracy	**-0.09 (-1.71)**	**0.14 (2.03)**	-0.08 (-1.05)	0.09 (1.15)	0.03 (0.32)
Penn emotion recognition Happy accuracy	**0.14 (1.80)**	0.08 (1.06)	-0.12 (-1.45)	0.04 (0.46)	0.08 (0.96)
Penn emotion recognition Neutral accuracy	0.02 (0.45)	0.03 (0.44)	**0.10 (1.74)**	0.04 (0.57)	0.04 (0.59)
Penn emotion recognition Sad accuracy	-0.04 (-0.71)	0.03 (0.34)	**-0.17 (-2.75)**	-0.01 (-0.14)	-0.01 (-0.10)
Penn emotion recognition Total accuracy	-0.06 (-1.11)	0.11 (1.65)	-0.08 (-1.13)	0.08 (1.16)	0.04 (0.48)
Penn emotion recognition Total RT	-0.03 (-0.46)	0.00 (0.00)	-0.09 (-1.07)	-0.06 (-0.57)	0.11 (1.05)
Hariri Emotion task accuracy	0.03 (0.44)	0.02 (0.28)	-0.08 (-0.96)	**-0.17 (-2.30)**	-0.01 (-0.13)
Hariri Emotion task RT	-0.03 (-0.50)	-0.05 (-0.66)	0.00 (-0.04)	**0.19 (1.90)**	0.00 (-0.05)
Anger affect (NIH)	**-0.44 (-3.68)**	**-0.33 (-4.08)**	-0.08 (-0.66)	0.00 (0.01)	0.02 (0.26)
Anger hostility (NIH)	**-0.32 (-3.19)**	**-0.17 (-2.28)**	-0.07 (-0.77)	0.01 (0.14)	-0.12 (-1.57)
Anger physical aggression (NIH)	**-0.25 (-3.71)**	-0.06 (-0.83)	0.02 (0.29)	0.05 (0.63)	0.03 (0.40)
Fear affect (NIH)	**-0.51 (-3.89)**	**-0.33 (-3.93)**	0.03 (0.27)	-0.08 (-0.74)	-0.12 (-1.24)
Fear somatic arousal (NIH)	**-0.44 (-3.75)**	**-0.22 (-2.50)**	-0.08 (-0.76)	0.14 (1.41)	-0.11 (-1.18)
Sadness (NIH)	**-0.40 (-3.50)**	**-0.32 (-3.73)**	-0.12 (-0.97)	0.01 (0.11)	-0.11 (-1.18)
Life satisfaction (NIH)	**0.35 (3.38)**	**0.20 (2.58)**	0.10 (1.04)	-0.08 (-0.95)	-0.01 (-0.16)
Meaning purpose (NIH)	**0.37 (4.62)**	0.09 (1.14)	0.10 (1.23)	0.06 (0.71)	-0.10 (-1.22)
Positive affect (NIH)	**0.34 (3.97)**	**0.20 (2.39)**	0.13 (1.39)	-0.07 (-0.74)	-0.10 (-1.17)
Friendship (NIH)	**0.20 (2.77)**	**0.23 (2.40)**	**0.22 (2.14)**	0.11 (1.14)	0.08 (0.87)
Loneliness (NIH)	**-0.34 (-3.71)**	**-0.22 (-2.69)**	-0.13 (-1.30)	-0.01 (-0.05)	-0.08 (-0.90)

Perceived hostility (NIH)	**-0.22 (-2.78)**	**-0.18 (-2.07)**	-0.10 (-1.06)	-0.03 (-0.25)	**-0.17 (-1.71)**
Perceived rejection (NIH)	**-0.31 (-3.75)**	**-0.17 (-1.77)**	**-0.20 (-2.19)**	0.02 (0.21)	-0.12 (-1.29)
Emotional support (NIH)	**0.25 (3.69)**	0.05 (0.54)	**0.18 (2.68)**	0.06 (0.72)	-0.02 (-0.18)
Instrumental support (NIH)	**0.16 (2.67)**	0.08 (1.20)	0.11 (1.60)	0.02 (0.24)	0.02 (0.27)
Perceived stress (NIH)	**-0.46 (-3.38)**	**-0.36 (-4.15)**	-0.07 (-0.53)	0.05 (0.39)	-0.09 (-0.90)
Self-efficacy (NIH)	**0.17 (2.80)**	**0.17 (2.05)**	0.06 (0.60)	0.15 (1.60)	0.08 (0.82)
Agreeableness (NEO-FFI)	**0.21 (2.22)**	0.00 (-0.03)	0.05 (0.52)	**-0.22 (-2.62)**	-0.01 (-0.13)
Openness experience (NEO-FFI)	**-0.13 (-2.08)**	-0.10 (-1.13)	**-0.22 (-2.94)**	0.03 (0.28)	-0.08 (-0.94)
Conscientiousness (NEO-FFI)	**0.28 (4.00)**	**0.34 (3.45)**	0.18 (1.41)	0.02 (0.20)	**-0.23 (-2.40)**
Neuroticism (NEO-FFI)	**-0.48 (-3.79)**	**-0.27 (-3.26)**	-0.05 (-0.47)	0.08 (0.79)	-0.08 (-0.92)
Extraversion (NEO-FFI)	**0.20 (2.97)**	**0.13 (1.74)**	**0.13 (1.72)**	0.08 (0.95)	-0.03 (-0.32)
Pain interference (NIH)	**-0.37 (-3.63)**	-0.09 (-1.08)	0.08 (0.86)	-0.07 (-0.70)	**-0.16 (-1.87)**
Lifetime alcohol dependence symptoms (SSAGA)	**-0.24 (-3.27)**	-0.09 (-1.02)	0.00 (0.03)	**-0.20 (-2.28)**	**-0.19 (-2.23)**
Lifetime alcohol abuse diagnosis (SSAGA)	**-0.18 (-2.08)**	**-0.14 (-1.93)**	0.04 (0.47)	0.04 (0.44)	-0.08 (-0.92)
Lifetime alcohol abuse symptoms (SSAGA)	**-0.25 (-3.11)**	-0.12 (-1.46)	0.06 (0.64)	-0.10 (-1.20)	-0.09 (-1.02)
Lifetime alcohol dependence diagnosis (SSAGA)	**-0.15 (-2.45)**	-0.09 (-1.09)	0.00 (-0.04)	**-0.26 (-3.16)**	**-0.17 (-1.89)**
Drinks per day past 12 months (SSAGA)	**-0.18 (-3.02)**	0.08 (1.16)	0.02 (0.22)	-0.03 (-0.39)	0.06 (0.81)
Frequency alcohol use past 12 months (SSAGA)	**0.10 (1.86)**	-0.02 (-0.23)	-0.05 (-0.75)	0.05 (0.60)	0.12 (1.61)
Frequency drunk past 12 months (SSAGA)	**0.16 (2.93)**	0.10 (1.24)	-0.13 (-1.62)	**0.14 (1.69)**	0.02 (0.29)
Age first alcohol use (SSAGA)	**0.22 (3.32)**	0.02 (0.27)	-0.07 (-0.98)	0.11 (1.50)	-0.05 (-0.58)
Drinks per day heaviest period (SSAGA)	**-0.14 (-2.20)**	0.05 (0.70)	0.11 (1.64)	-0.01 (-0.10)	0.02 (0.30)
Frequency alcohol use heaviest period (SSAGA)	**0.10 (1.77)**	0.02 (0.37)	-0.01 (-0.15)	0.07 (1.04)	0.07 (0.99)
Frequency drunk heaviest period (SSAGA)	**0.12 (1.69)**	0.07 (0.96)	-0.06 (-0.83)	0.03 (0.31)	0.02 (0.23)
History of smoking (SSAGA)	**-0.15 (-2.18)**	-0.05 (-0.76)	0.02 (0.34)	0.01 (0.07)	-0.06 (-0.79)
Currently smoking (SSAGA)	**-0.16 (-1.99)**	-0.01 (-0.16)	0.13 (1.46)	0.13 (1.39)	**-0.19 (-2.26)**
Times used nonmarijuana illicit drugs (SSAGA)	**-0.21 (-3.05)**	**-0.20 (-2.60)**	0.11 (1.19)	-0.04 (-0.44)	0.03 (0.38)
Times used cocaine (SSAGA)	**-0.21 (-2.87)**	-0.10 (-1.50)	0.03 (0.39)	-0.01 (-0.08)	0.00 (0.02)
Times used hallucinogens (SSAGA)	**-0.11 (-1.77)**	**-0.21 (-3.00)**	0.06 (0.61)	-0.04 (-0.45)	-0.03 (-0.39)
Times used opiates (SSAGA)	**-0.19 (-2.73)**	**-0.20 (-2.53)**	0.12 (1.14)	0.01 (0.14)	-0.08 (-0.90)
Times used sedatives (SSAGA)	**-0.27 (-3.37)**	**-0.13 (-1.82)**	0.02 (0.18)	0.02 (0.28)	-0.02 (-0.30)
Times used stimulants (SSAGA)	-0.10 (-1.64)	**-0.12 (-1.69)**	0.01 (0.19)	-0.04 (-0.49)	0.03 (0.41)
Times used marijuana (SSAGA)	**-0.17 (-2.56)**	-0.08 (-1.15)	0.08 (1.09)	-0.07 (-0.92)	-0.05 (-0.67)
Marijuana dependence (SSAGA)	-0.09 (-1.38)	**-0.28 (-3.92)**	0.06 (0.60)	-0.04 (-0.47)	0.05 (0.59)

Table C

Cross-validated canonical correlation analysis. Significant p-values are indicated in bold.

	In-sample correlation		Out-of-sample correlation		
	Whole set	Permuted p	Training sets (mean [range] across folds)	Test sets (mean [range] across folds)	Permuted p (mean [range] across folds)
LC1	*r* = 0.69	***p* = 0.0002**	r=0.71 [0.71-0.72]	*r* = 0.49 [0.44-0.53]	***p* = 0.001 [0.001-0.001]**
LC2	*r* = 0.53	***p* = 0.0002**	r=0.57 [0.55-0.58]	*r* = 0.19 [0.11-0.26]	***p* = 0.039 [0.001-0.155]**
LC3	*r* = 0.49	***p* = 0.0002**	r=0.54 [0.53-0.55]	*r* = 0.12 [0.06-0.29]	*p* = 0.209 [0.001-0.398]
LC4	*r* = 0.44	***p* = 0.0002**	r=0.49 [0.47-0.51]	*r* = 0.01 [-0.05-0.07]	*p* = 0.497 [0.169-0.873]
LC5	*r* = 0.42	***p* = 0.0002**	r=0.46 [0.44-0.48]	*r* = 0.06 [-0.09-0.24]	*p* = 0.336 [0.017-0.787]

Table D

Post-hoc associations between sleep (or biopsychosocial; BPS) composite scores and sociodemographic measures, as well as physical and mental health measures. Associations using continuous measures (e.g., age, education years) were tested with Pearson correlations, while categorical measures (e.g., sex, race) were assessed with t-tests or analyses of variance. Associations indicated in bold were found to be statistically significant after FDR correction (q < 0.05). Tests with <5 subjects in a category were not computed because of too little variance.

		LC1		LC2		LC3		LC4		LC5	
		Sleep scores	BPS scores	Sleep scores	BPS scores	Sleep scores	BPS scores	Sleep scores	BPS scores	Sleep scores	BPS scores
Age	*r*	0.02	0.01	-0.01	-0.02	-0.04	-0.06	**0.08**	**0.10**	0.03	-0.03
	p	0.492	0.851	0.790	0.621	0.308	0.085	**0.020**	**0.007**	0.398	0.478
Sex	*T*	-0.86	-0.86	0.52	0.51	-0.92	-0.92	1.75	1.88	**-2.88**	**-4.35**
	p	0.388	0.388	0.601	0.613	0.359	0.357	0.081	0.061	**0.004**	**0.000**
Education	*r*	**-0.11**	**-0.14**	**0.10**	**0.11**	0.00	-0.03	**-0.07**	**-0.12**	**-0.11**	**-0.22**
	p	**0.003**	**0.000**	**0.007**	**0.002**	0.918	0.379	**0.048**	**0.001**	**0.002**	**0.000**
Race	*F*	0.69	1.07	0.24	1.32	**3.76**	1.19	**2.44**	**3.22**	**2.67**	**4.79**
	p	0.631	0.373	0.945	0.254	**0.002**	0.312	**0.033**	**0.007**	**0.021**	**0.000**
Ethnicity	*F*	2.11	**5.29**	0.25	1.79	0.83	1.57	0.66	1.72	0.50	1.36
	p	0.122	**0.005**	0.780	0.168	0.436	0.209	0.517	0.180	0.608	0.257
Household income	*F*	**2.35**	**3.82**	0.89	**2.69**	0.52	0.55	0.83	0.89	1.80	0.88
	p	**0.022**	**0.000**	0.513	**0.009**	0.817	0.798	0.565	0.512	0.083	0.518
Employment status	*F*	1.89	2.42	**3.95**	1.34	0.23	1.08	2.96	**4.41**	0.16	0.17
	p	0.152	0.090	**0.020**	0.262	0.796	0.342	0.052	**0.013**	0.849	0.840
School status	*F*	-0.10	-0.18	**-2.23**	-1.46	-0.80	1.18	**-2.11**	-0.86	0.42	1.19
	p	0.923	0.857	**0.026**	0.146	0.424	0.237	**0.035**	0.388	0.673	0.236
Relationship status	*F*	0.43	1.05	-0.06	0.23	1.16	0.64	-0.15	0.78	0.68	-0.40
	p	0.665	0.294	0.955	0.816	0.247	0.523	0.882	0.437	0.495	0.689
BMI	*r*	0.04	0.03	-0.05	-0.02	**-0.11**	-0.03	**0.08**	0.05	0.05	-0.01
	p	0.261	0.463	0.151	0.603	**0.003**	0.394	**0.024**	0.196	0.179	0.678
Hematocrit	*r*	0.05	0.02	-0.03	0.01	-0.03	-0.04	-0.02	0.01	-0.02	0.05
	p	0.150	0.499	0.423	0.819	0.461	0.214	0.514	0.831	0.652	0.142
Blood pressure	*F*	0.87	0.57	1.09	0.46	0.86	0.53	1.57	0.46	0.82	0.48
	p	0.483	0.683	0.360	0.768	0.485	0.714	0.179	0.767	0.514	0.747
Maternal history of depression	*t*	-1.50	-1.76	-1.54	-1.39	-0.36	0.78	1.22	0.27	-0.20	-1.75
	p	0.135	0.080	0.123	0.166	0.716	0.438	0.222	0.791	0.840	0.080
Paternal history of depression	*t*	-1.44	**-2.01**	**-2.24**	-0.80	0.54	-1.00	-0.76	1.48	-1.18	-1.65
	p	0.150	**0.044**	**0.025**	0.425	0.587	0.320	0.447	0.139	0.238	0.100
Maternal history of BD	*t*	-0.18	0.79	-1.37	-0.64	-0.17	-1.03	-1.84	-0.35	-1.17	-1.31
	p	0.860	0.428	0.173	0.522	0.868	0.303	0.066	0.726	0.242	0.190
Paternal history of BD	*t*	-0.39	-0.23	-1.21	-0.54	1.93	1.69	-1.05	1.85	0.42	-0.76
	p	0.694	0.815	0.228	0.590	0.055	0.091	0.293	0.065	0.677	0.450
Maternal history of anxiety	*t*	-1.82	-0.86	-1.38	**-2.38**	-0.25	-1.25	0.62	0.34	0.05	-1.79
	p	0.069	0.391	0.167	**0.017**	0.801	0.213	0.532	0.735	0.959	0.074
Paternal history of anxiety	*t*	-0.67	0.38	-1.54	-0.52	1.13	0.39	0.85	**2.22**	-0.92	0.07
	p	0.505	0.708	0.124	0.606	0.261	0.693	0.397	**0.027**	0.357	0.948
	t	-0.91	-0.76	0.91	0.59	-0.22	0.92	0.02	-1.97	-1.47	-1.70

Maternal history of drug or alcohol problems	*p*	0.366	0.448	0.363	0.556	0.829	0.357	0.987	0.050	0.143	0.089
Paternal history of drug or alcohol problems	*t*	-0.61	-1.05	-0.90	**-2.21**	1.08	1.57	-0.60	-0.16	-0.11	-0.95
	p	0.542	0.293	0.369	**0.028**	0.283	0.117	0.549	0.870	0.916	0.343
Maternal history of SZ	*t*	-	-	-	-	-	-	-	-	-	-
	p	-	-	-	-	-	-	-	-	-	-
Paternal history of SZ	*t*	-	-	-	-	-	-	-	-	-	-
	p	-	-	-	-	-	-	-	-	-	-
Maternal history of Alzheimer’s disease or dementia	*t*	-	-	-	-	-	-	-	-	-	-
	p	-	-	-	-	-	-	-	-	-	-
Paternal history of Alzheimer’s disease or dementia	*t*	-	-	-	-	-	-	-	-	-	-
	p	-	-	-	-	-	-	-	-	-	-
Maternal history of Parkinson’s disease	*t*	-	-	-	-	-	-	-	-	-	-
	p	-	-	-	-	-	-	-	-	-	-
Paternal history of Parkinson’s disease	*t*	-	-	-	-	-	-	-	-	-	-
	p	-	-	-	-	-	-	-	-	-	-
Maternal history of Tourette’s syndrome	*t*	-	-	-	-	-	-	-	-	-	-
	p	-	-	-	-	-	-	-	-	-	-
Paternal history of Tourette’s syndrome	*t*	-	-	-	-	-	-	-	-	-	-
	p	-	-	-	-	-	-	-	-	-	-

Table E

Correlations between sleep (or biopsychosocial) loadings in control analyses with those from the original analysis. *Note that LC1, LC2 and LC5 are inverted in our manuscript with regards to their interpretation, as the directionality of CCA loadings and scores is arbitrary.*

		Not regressing out confounds	Excluding participants positive for substance use	Excluding physical health variables *	Excluding demographic variables**	Quantile normalization on biopsychosocial / sleep data	PCA on biopsychosocial data	Female participants only	Male participants only
LC1	Sleep loadings	-1.00	-0.97	-1.00	-1.00	0.62	0.79	0.88	0.90
	Biopsychosocial loadings	-1.00	-0.99	-1.00	-1.00	-0.16	0.99	0.96	0.96
LC2	Sleep loadings	-1.00	-0.96	-0.99	-1.00	0.95	0.66	-0.80	-0.86
	Biopsychosocial loadings	-0.99	-0.95	-1.00	-1.00	0.97	0.74	-0.90	-0.88
LC3	Sleep loadings	1.00	0.93	0.99	1.00	-0.71	0.60	-0.60	-0.73
	Biopsychosocial loadings	0.99	0.85	0.97	0.99	-0.58	0.56	-0.54	-0.47
LC4	Sleep loadings	0.98	0.81	0.98	0.98	-0.95	-0.86	-0.69	0.64
	Biopsychosocial loadings	0.94	0.75	0.99	0.97	-0.89	-0.85	-0.22	0.63
LC5	Sleep loadings	-0.94	-0.59	-0.87	0.95	0.53	-0.73	-0.49	0.54
	Biopsychosocial loadings	-0.94	-0.58	-0.91	0.95	0.67	-0.76	-0.57	0.13

* BMI, Hematocrit, Blood pressure

** Employment status, Household income, In school, Relationship status

Supplementary Figures

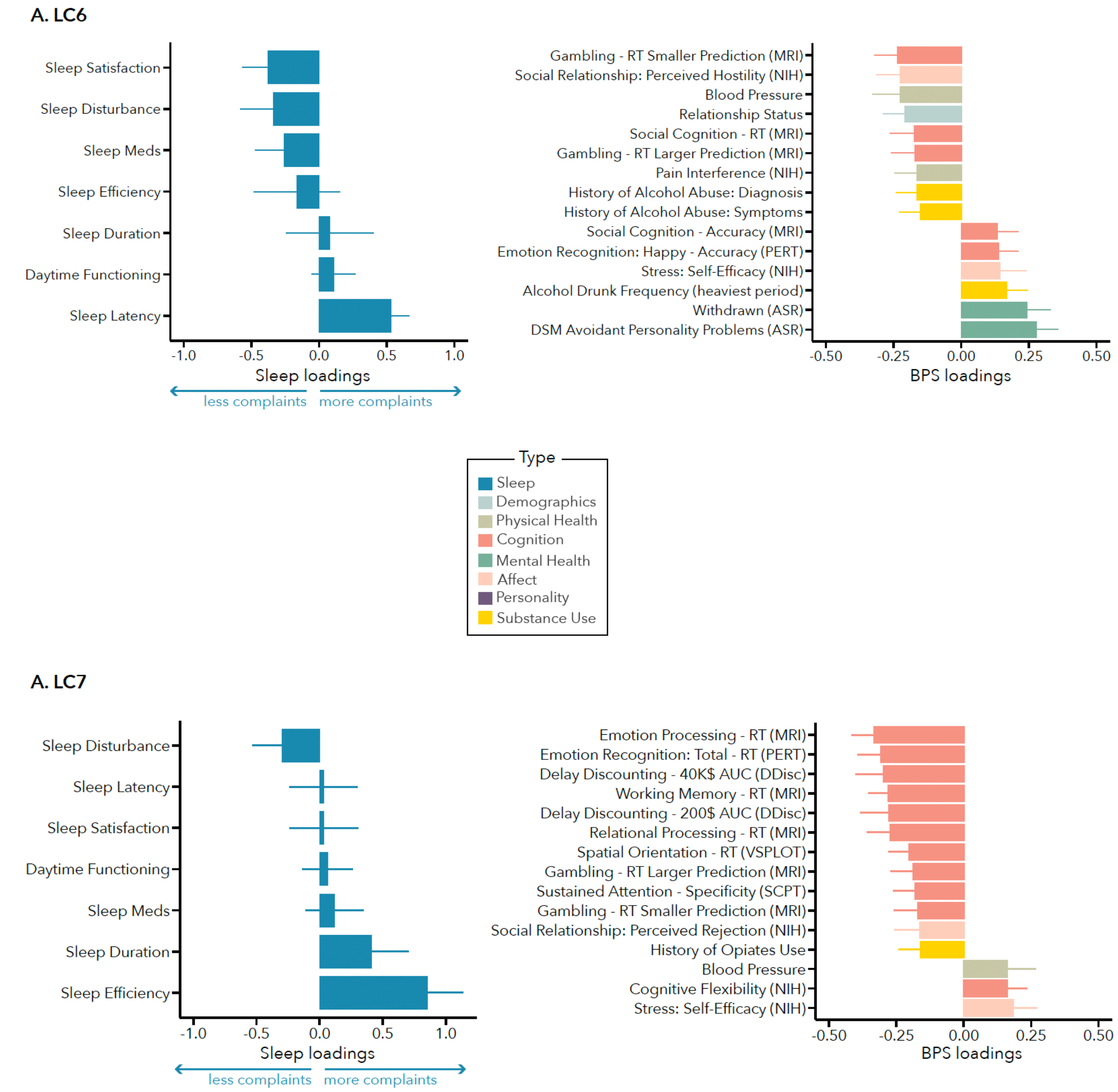

Figure A

Latent Components LC6 and LC7

(A) Sleep loadings (left) and top 15 strongest biopsychosocial (BPS) loadings (right) for LC6. (B) Sleep loadings (left) and top 15 strongest biopsychosocial (BPS) loadings (right) for LC7.

Positive values on sleep (blue) loadings indicate worse outcomes while positive values on biopsychosocial loadings reflect higher magnitude on these measures. Error bars indicate bootstrapped-estimated confidence intervals (i.e., standard deviation) and measures in bold indicate statistical significance. ***See S1 Data for underlying data.***

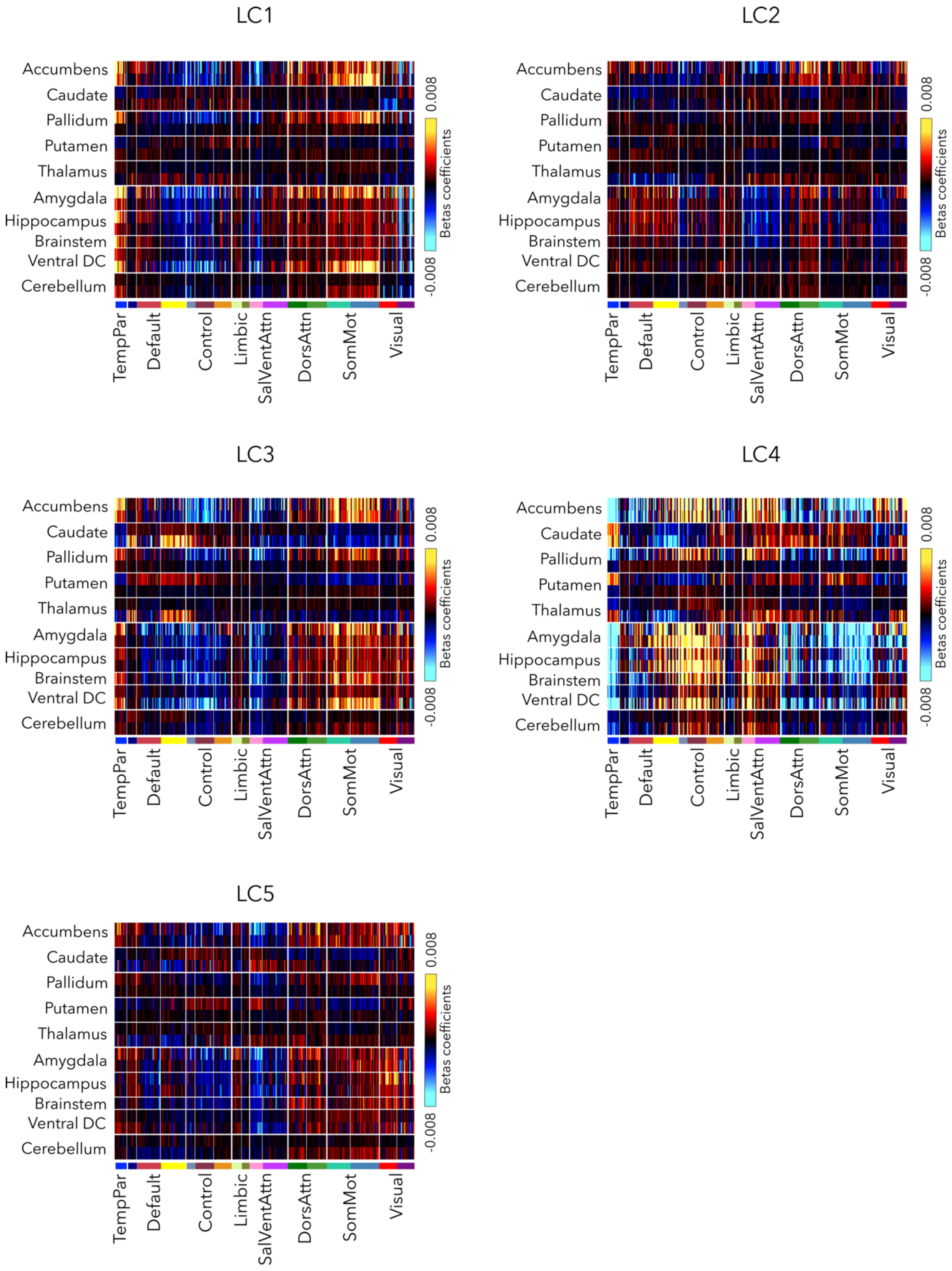

Figure B

Post-hoc associations between CCA mean composite scores and RSFC, highlighting beta coefficients between subcortical regions and cortical networks.

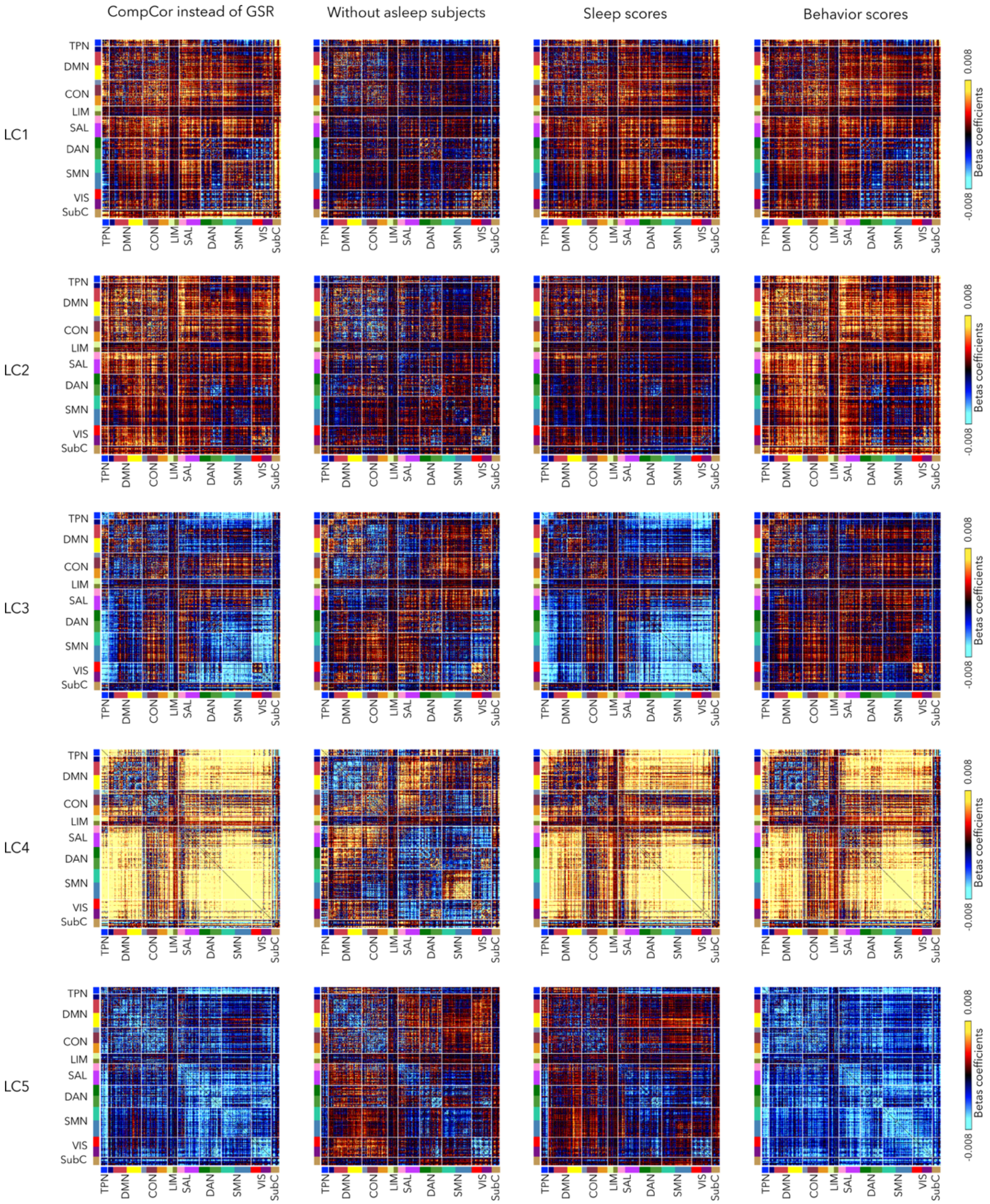

Figure C

Control analyses for post-hoc associations with RSFC. (**Left panel**) GLM analysis using RSFC data that underwent CompCor instead of global signal regression. (**Middle left panel**) GLM analysis after excluding subjects that likely fell asleep in the scanner (N=100). (**Middle right panel**) GLM analysis between RSFC and *sleep* composite scores (instead of averaged composite scores). (**Right panel**) GLM analysis between RSFC and *biopsychosocial* composite scores (instead of averaged composite scores).

Abbreviations: CON=Executive control network, DAN=Dorsal attention network, DMN=Default mode network, LIM=Limbic network, SAL=Salience/Ventral attention network, SMN=Somatosensory-motor network, SubC=Subcortical regions, TPN=Temporoparietal network, VIS=Visual network.

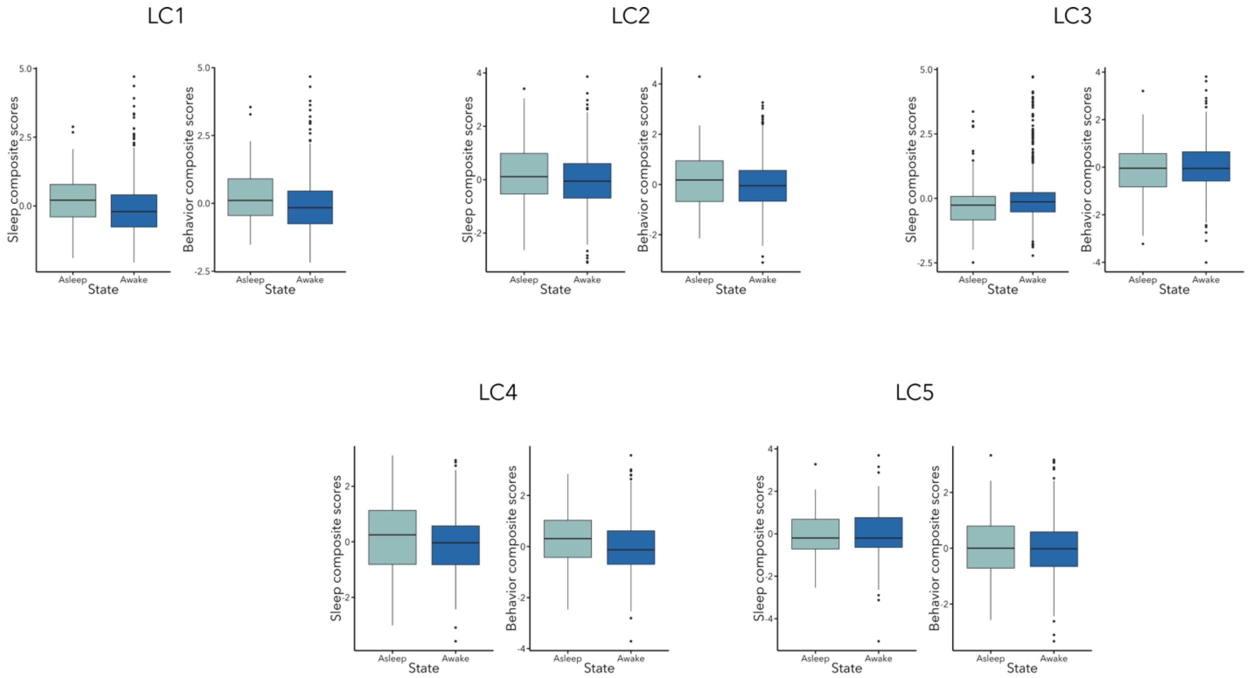

Figure D

Post-hoc t-tests assessing differences in sleep (or biopsychosocial) composite scores between participants who likely stayed awake (N=623) in the scanner vs. those who likely fell asleep in the scanner (N=100). **See S1 Data for underlying data.**

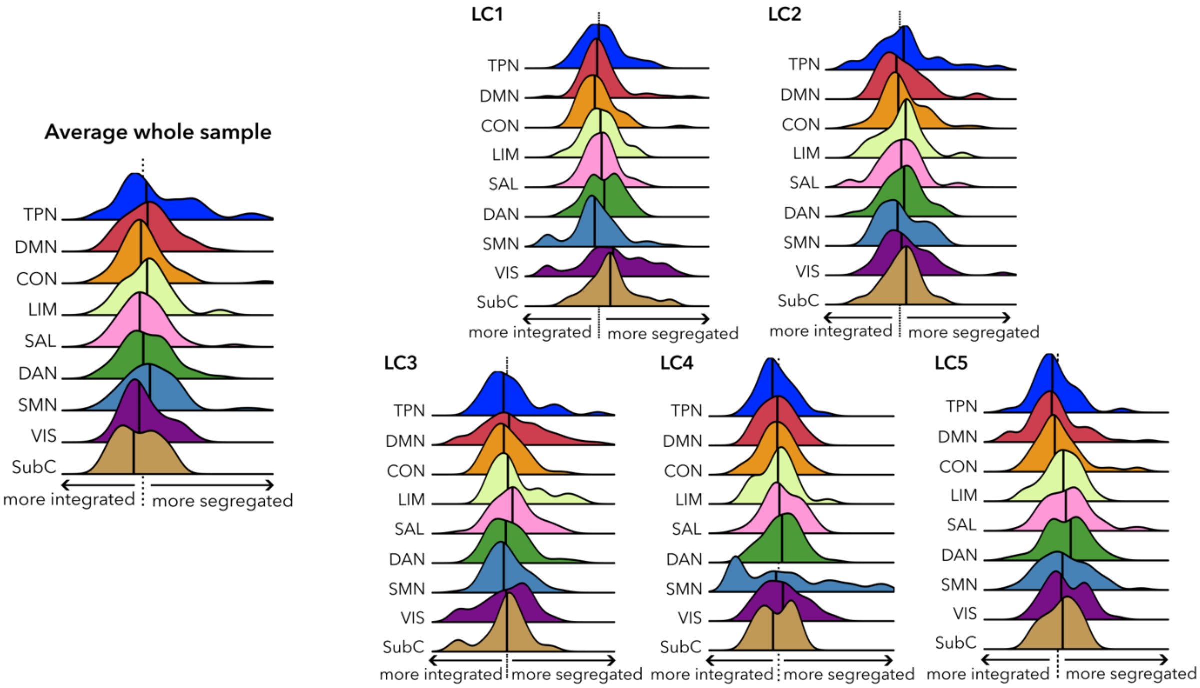

Figure E

Distribution of the segregation and integration ratio on the average RSFC matrix of the whole sample compared to integration and segregation measures per LC. The dashed line indicates the median of all parcels, and the bold black lines represent the median for each network.

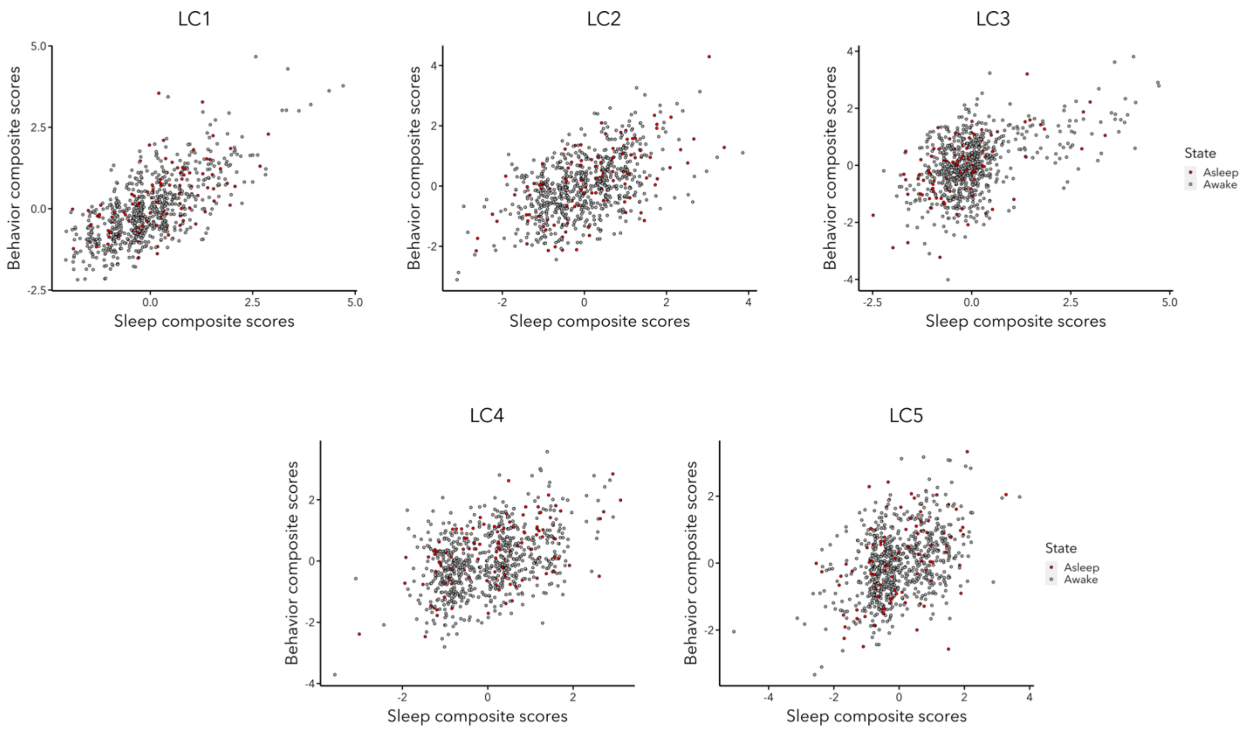

Figure F

Scatterplots showing the distribution of sleep and biopsychosocial composite scores of participants who likely fell asleep in the scanner (in red) compared to those that likely stayed awake (in grey).

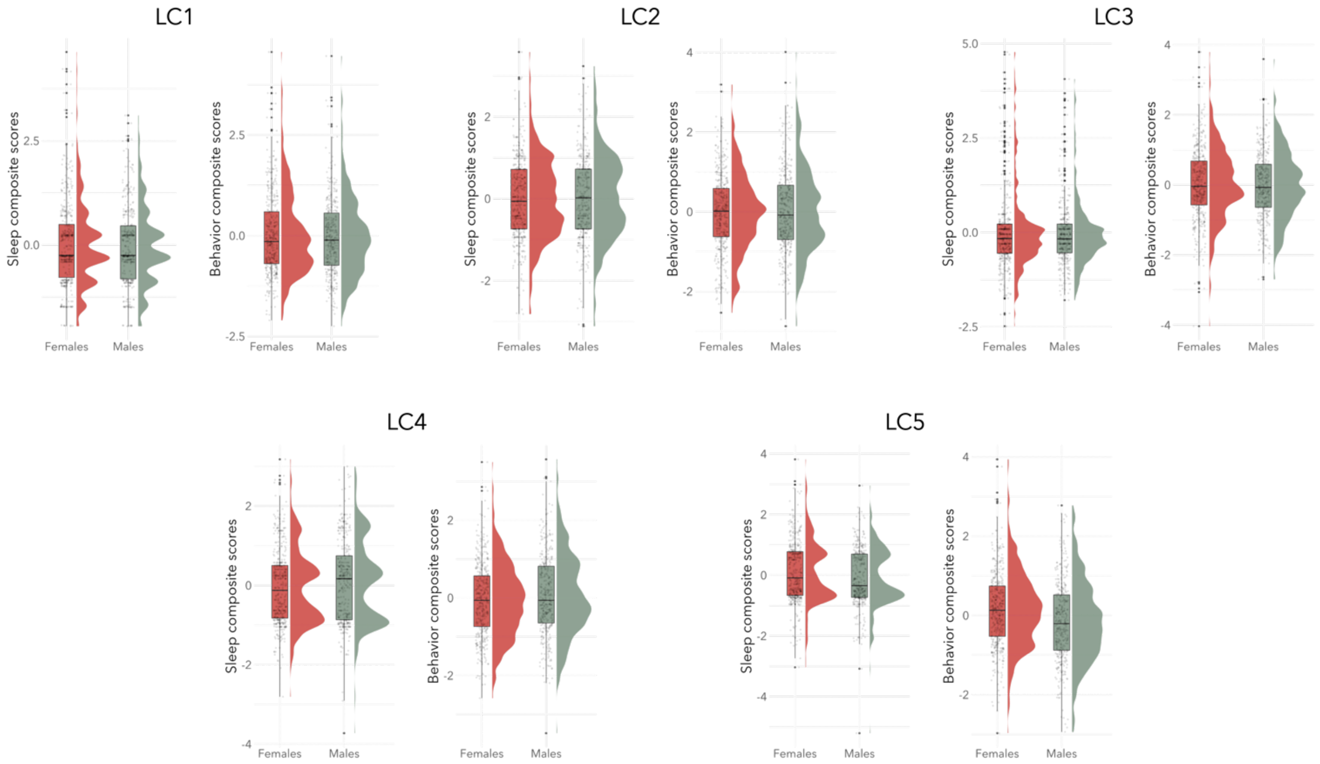

Figure G

Post hoc analyses testing for sex differences in the sleep and behavior composite scores between male (green) and female (red) participants. Male participants had significantly lower sleep ($t=-2.88$, $p=0.004$) and biopsychosocial ($t=-4.35$, $p<0.001$) scores for LC5 compared to female participants. **See S1 Data for underlying data.**
