# Supplementary material for: Identification of five sleep-biopsychosocial profiles with specific neural signatures linking sleep variability with health, cognition, and lifestyle factors
Source: PLoS Biol. 2025 Oct 7;23(10):e3003399. doi: 10.1371/journal.pbio.3003399 (PMC12503254; doi:10.1371/journal.pbio.3003399)
Supplement: Attachment — Submitted filename: response_to_reviewer.pdf [file pbio.3003399.s005.pdf]

We thank the reviewers for their time in reviewing our work and providing feedback. Please find our responses written **in blue**. Changes in the manuscript are provided in blue and *italics*.

Reviewer #1: In this work, the authors used CCA in the HCP dataset to derive latent components describing the multivariate relations between sleep and cognition/psychopathology factors. They then related these latent components to the brain functional connectivity profiles of the participants. I generally like this paper and do not have many comments. I did want to see some discussion on the following:

1. Even though the sample size is greater than the number of variables (n > p) here, it is not more than 10 times larger (my own simulations have shown n > 12*p works well for CCA). I was wondering if some dimension reduction might be a good further sensitivity analysis to perform.

We thank the reviewer for suggesting this important sensitivity analysis. We applied principal component analysis (PCA) to the biopsychosocial variables and re-computed a CCA between the 7 sleep variables and 67 PCA scores that explained 90% of the variance among the 118 biopsychosocial variables. Just like in the original CCA analysis, we found all 7 LCs to be significant (ps < 0.001). After matching the LCs between the two models using the Hungarian algorithm, we compared the loadings from both models and found strong correlations between the LC loadings from the original model and the PCA-CCA model, as shown in the following table (note that the sign of the CCA is arbitrary, e.g.,-0.9 and 0.9 are to be interpreted similarly):

	Correlation with original sleep loadings	*Correlation with original biopsychosocial loadings*
LC1	0.7924	0.9912
LC2	0.6589	0.7378
LC3	0.5957	0.5586
LC4	-0.8606	-0.8451
LC5	-0.7307	-0.7633
LC6	-0.9387	-0.8954
LC7	-0.8268	-0.6902

We now report this sensitivity analysis in the Methods and Results section, as well as in the Supplemental Results.

<u>Methods</u> – Page 15

"Sixth, to test the stability of our LCs, we re-computed the CCA after reducing the dimensionality of the biopsychosocial variables and using the principal components that explained 90% of the variance among the 118 biopsychosocial variables."

Results – Page 8 and Supplemental Results (Table S5)

"Second, we re-computed the CCA analysis after: (i) applying quantile normalization on sleep and biopsychosocial measures; (ii) excluding participants that had tested positive for any substance on the day of the MRI; (iii) excluding physical health measures (i.e., body mass index, hematocrit, blood pressure), (iv) excluding sociodemographic variables (i.e., employment status, household income, school status, relationship status) from the biopsychosocial matrix; (v) *after using PCA to reduce the dimensionality of the biopsychosocial variables;* and (vi) after considering only female (or male) participants in the CCA."

2. The dominance of the LC1 over the other LCs in the attributed covariance should be brought to the attention of the readers more and discussed a bit (and maybe temper some of the conclusions about the additional LCs).

We thank the reviewer for their feedback about the importance of the LCs. We have restructured the whole discussion to emphasise the discussion around LC1 and the fact that it captured most of the covariance with biopsychosocial measures, and we reduced the discussion about LCs 2-5, although we think they might be informative in describing covariance patterns that are either likely observed in a fraction of the participants, or in all or most participants but with less prominence.

Results – Page 4

"While LC1 accounted for a substantial amount of covariance between sleep and biopsychosocial measures, LCs 2-5 highlighted distinct covariance patterns that were driven by specific sleep dimensions, probably representative of only a fraction of the participants, or present in all or most participants but with less prominence."

Discussion – Page 8-10

We added more info on LC1 while reducing the paragraph on the other LCs

"We found that the first profile explained most of the variance and reflected general psychopathology (or p factor) associated with general poor sleep (LC1)."

"LC1 overwhelmingly explained 88% of the variance in a sample of healthy adults, highlighting the reciprocal relation between sleep and psychopathology and how sleep may be of utmost importance for both the prevention and treatment of mental disorders. LC1 reflected general psychopathology associated with overall poor sleep akin to insomnia complaints (i.e., difficulties falling asleep, maintaining sleep, sleep dissatisfaction, insufficient sleep). While we could not assess the presence of insomnia disorder based on clinical diagnostic criteria (54,55) or the chronicity of sleep complaints (i.e., PSQI captures sleep complaints only for the previous month)(39), there is a body of evidence of a reciprocal relationship between reports of poor sleep or insomnia complaints and psychopathology(56). Overall, poor sleep is not only a risk factor but also a co-morbid condition and transdiagnostic symptom for many mental disorders(57). When sleep is disrupted, it also contributes to the dysregulation of multiple neurobiological mechanisms related to emotional regulation and psychopathology(58). The strong co-morbidity between poor sleep and psychopathology is evident from a young age, as suggested by a recent study that used a similar

data-driven approach (i.e., CCA) to assess associations between parent-reported sleep disturbances and a broad set of psychological, demographics and cognitive variables in a large sample of 9-10 year old children(59). The study reported a very similar latent component linking general poor sleep to general psychopathology, and that covariance pattern was replicated after a 2-year follow-up, suggesting the robustness of this association over time.

Although LC1 captured a large amount of covariance between sleep and biopsychosocial measures, LCs 2-5 showed covariance patterns that were characterised by specific sleep dimensions, which likely described associations seen in a fraction of the participants; alternatively, these associations might also be present in all or most participants, but with less prominence."

3. Discussion paragraph 5: A recent preprint relates sleep and biopsychosocial factors to somatomotor disconnect in the ABCD dataset [1]. I was wondering if the SMN being more integrated than segregated in the LC1 in this study is the opposite of the findings from the ABCD in [1]? If that is the case, please discuss this.
[1] Michael, C., Taxali, A., Angstadt, M., McCurry, K. L., Weigard, A., Kardan, O., ... & Sripada, C. (2024). Somatomotor disconnection links sleep duration with socioeconomic context, screen time, cognition, and psychopathology. bioRxiv, 2024-10.

We thank the reviewer for drawing our attention to this interesting preprint. Michael and colleagues found a significant link between reduced sleep duration and disconnection of the somatomotor network (i.e., increased within-module degree and reduced participation coefficient). In our study, the first latent component (LC1) reflects general poor sleep (including sleep duration, sleep onset, sleep quality, etc) and psychopathology, whereas LC4 reflects sleep duration and cognition, which might share more similarity with the suggested paper. LC1 was associated with more variability in segregation and integration in the visual network, while LC4 had a similar variability in segregation and integration in the somatomotor network to that of Michael and colleagues. We did find a localised segregation of part of the somatomotor network associated with LC4, which is similar to what is reported in that study.

We would like to highlight however that there are some striking differences between that study and ours, notably the age of participants (11-12 years vs 22-36 years here) and the calculation of sleep duration (a latent component comprised of self-report, chronotype and Fitbit data vs. self-report alone here). As such, this may explain some differences in findings. Nonetheless, we have referenced this article in our introduction and discussion:

<u>Introduction</u> - Page 3
"Such unidimensional studies have independently linked insufficient or poor sleep to a wide range of negative outcomes separately, including cognitive difficulties(11,12), *brain connectivity changes(13–17),* decreased physical health(7,18), poor mental health and well-being(8,19), as well as increased risks for cardiovascular disease(7,20,21), neurodegenerative disease(22,23) and psychiatric disorders(8,24)."

<u>Discussion</u> – Page 10

“Interestingly, the strong RSFC patterns associated with LC4 showed a global increase in connectivity, alongside localised segregation of part of the somatomotor network. *Similar patterns have been reported in neuroimaging studies of experimental acute total sleep deprivation(74,75) and were recently found to be associated with sleep duration in adolescents(16). This suggests that LC4 may reflect underlying sleep debt in the general population”*

4. Discussion paragraphs 2 and later 6: Some more recent works to potentially discuss if relevant: There is recent work relating sleep disturbances to different psychosocial domains in the ABCD data using CCA [1], and work using both HCP and ABCD datasets on relations between brain functional profiles and sleep duration [2]
[1] McCurry, K. L., Toda-Thorne, K., Taxali, A., Angstadt, M., Hardi, F. A., Heitzeg, M. M., & Sripada, C. (2024). Data-driven, generalizable prediction of adolescent sleep disturbances in the multisite Adolescent Brain Cognitive Development Study. Sleep, 47(6).
[2] Mummaneni, A., Kardan, O., Stier, A. J., Chamberlain, T. A., Chao, A. F., Berman, M. G., & Rosenberg, M. D. (2023). Functional brain connectivity predicts sleep duration in youth and adults. Human Brain Mapping, 44(18), 6293-6307.

Thank you for suggesting the addition of these articles to the manuscript. We have added them to the introduction and discussion.

Introduction – Page 3
“Such unidimensional studies have independently linked insufficient or poor sleep to a wide range of negative outcomes separately, including cognitive difficulties(11,12), *brain connectivity changes(13–17),* decreased physical health(7,18), poor mental health and well-being(8,19), as well as increased risks for cardiovascular disease(7,20,21), neurodegenerative disease(22,23) and psychiatric disorders(8,24).”

Discussion – Page 9
“The strong co-morbidity between poor sleep and psychopathology is evident from a young age, as suggested by a recent study that used a similar data-driven approach (i.e., CCA) to assess associations between parent-reported sleep disturbances and a broad set of psychological, demographics and cognitive variables in a large sample of 9-10 year old children(59). The study reported a very similar latent component linking general poor sleep to general psychopathology, and that covariance pattern was replicated after a 2-year follow-up, suggesting the robustness of this association over time.”

Reviewer #2 (Kimberly Cote): The authors report on a multivariate data-driven analysis of the role of sleep quality and duration in biopsychosocial factors in a large sample of healthy young adults (n=770) from a publicly available dataset. Sleep and most outcome measures are based on self-report. Through canonical correlation analysis, the authors arrive at 5 latent components (LCs) that summarize the relationships between the subcomponents of the PSQI and 118 outcome measures.

As a second aim, the authors describe the resting-state brain activity exhibited by the 5 LC profiles.

This is an interesting, data-rich, and well written paper offering some novel data. Examination of relationship between multiple variables in large samples is greatly needed to understand the complexity of the relationship between sleep and waking cognitive function and (mental) health. While self-report measures (particularly the PSQI for sleep quality) have some limitations, this is acknowledged by the authors, and the paper paves the way for future research to examine the LCs reported here using more objective measures of sleep. Additionally, while there are differences between objective and subjective measures of sleep, and the magnitude of this difference varies across individuals, there is still something important for health captured by the variable of one's perception of their sleep.

Sex differences are either controlled or investigated in the various analyses reported in the paper. For instance, it is reported that for LC5, higher sleep and biopsychosocial composite scores were found in females (page 7). More specific interpretation of the findings would be helpful. Is this consistent with the literature generally showing sleep is poorer and psychopathology more prevalent in females? Did the authors run the analyses in the females alone, and then the males alone? Are all 5 LCs seen within each sex? And does the strength of associations have a different pattern in males and females? For LC5, the authors explain that sleep disturbance may be due to a variety of factors fragmenting sleep, such as "difficultly maintaining optimal body temperature" (page 9). It would therefore be interesting in future research to see if this profile fits menopausal women in an older sample.

We thank the reviewer for their positive feedback on the manuscript and for bringing these biological sex-related questions to our attention. In our main analysis, sex differences were indeed regressed out from the data before the CCA was computed (alongside age and education level). We did, however, investigate sex differences in a sensitivity analysis that did not control for these covariates, and found that female participants had higher sleep and biopsychosocial composite scores for LC5 only compared to male participants (**Table S4**, **Figure S7**). Sleep and biopsychosocial scores in male vs. female participants are shown in **Figure S7**.

We have revised the Methods section to add details about the covariates that were adjusted for in the CCA.

<u>Methods</u> – Page 13
*"*Canonical Correlation Analysis (CCA)(100,101), a multivariate data-driven approach, was applied to the sleep and biopsychosocial measures, *after regressing out the effects of age, sex, and education from both the sleep and biopsychosocial variables."*

We also mentioned the sex differences in LC5 in the discussion:

<u>Discussion</u> - Page 9-10
"Interestingly, biological sex differences emerged only in LC5, with female participants showing higher sleep and biopsychosocial scores than male participants. Such differences often arise at

puberty, with female participants reporting more sleep fragmentation (i.e., awakenings, time spent awake in the middle of the night)(69) and higher rates of depression and anxiety across the lifespan (i.e., reproductive events) (70) compared to male participants."

While Reviewers 1 and 3 asked to focus on LC1 and tone down our discussion of LCs 2-5, we do agree with the reviewer's comments on disrupted thermoregulation and the possible presence of LC5 more predominantly in older females. The present cohort is composed of young adults, but running a similar analysis in an older adult cohort might show different insights, especially when considering biological sex differences. We argue about the importance of further investigating sex differences in sleep and biopsychosocial associations in the discussion.

Discussion - Page 10 and 12
"While there was no sex difference in the other LCs, the sex specificity of LC5 highlights the meaningful interplay between biological sex and individual differences in sleep disturbances and mental health"

"Future investigations into how the multifaceted relationships between sleep and biopsychosocial factors differ or change according to age, sex, and other demographics would likely benefit from data-driven approaches."

Additionally, we ran two separate CCA analyses in respectively only female and only male participants. In the model with female participants only (N=414), all 7 LCs were significant (all ps=0.0002 except LC7 p=0.0021). The first two LCs are highly similar to the first two LCs from the original model, while the rest of the LCs show low to moderate correlation to the original LCs (as shown in the table below).

	Correlation with original sleep loadings	*Correlation with original biopsychosocial loadings*
LC1	0.8769	0.9627
LC2	-0.7952	-0.9010
LC3	-0.6031	-0.5361
LC4	-0.6875	-0.2212
LC5	-0.4902	-0.5750
LC6	0.5570	0.5546
LC7	-0.8781	-0.6187

In the CCA with male participants only (N=356), all LCs except LC7 were found to be significant (all ps < 0.001 except LC7 p=0.06). Again, the first two LCs are highly similar, while the rest of the LCs is low to moderately similar to the original LCs.

	Correlation with original sleep loadings	*Correlation with original biopsychosocial loadings*
LC1	0.8985	0.9598
LC2	-0.8565	-0.8800
LC3	-0.7335	-0.4743
LC4	0.6450	0.6329
LC5	0.5385	0.1254
LC6	0.6554	0.2565
LC7	0.8714	0.5074

While LC1 and LC2 appear to be robust across participants of both sexes, LCs 3-7 appear to be more unstable within separate samples of males and female participants, probably due to the smaller sample size (i.e., cut in half compared to the original CCA).

We added this new analysis in the Supplemental Results:
"***Control per biological sex analyses***
Additionally, we ran two separate CCA in respectively only female and only male participants.
In the model with female participants only (N=414), all 7 LCs were significant (all ps=0.0002 except LC7 p=0.0021). The first two LCs are highly similar to the first two LCs from the original model, while the rest of the LCs show low to moderate correlation to the original LCs (Table S5). In the CCA with male participants only (N=356), all LCs except LC7 were found to be significant (all ps < 0.001 except LC7 p=0.06). Again, the first two LCs are highly similar, while the rest of the LCs is low to moderately similar to the original LCs (Table S5). The sex specificity highlights the meaningful interplay between biological sex and individual differences in sleep and biopsychosocial factors, however this could also be due to the reduced sample size in each sex-specific model."

LC2 is a group with high psychopathology, but healthy sleep. The authors have interpreted this as possibly reflecting a resilient group whose sleep is not impacted by the stressors of psychopathology. This is an interesting individual difference hypothesis, one that could be investigated in future research to examine possible factors explaining the resilience. However, there are other possible explanations. This group could lack insight into their sleep disturbance, or not care enough to report it as a major concern (particularly relative to other concerns that may have more weight for them). Follow-up objective studies can address this. Another possibility is this group may not have developed the associated sleep disturbance yet- is there data in this sample to consider the length of time that symptoms of psychopathology have been problematic for individuals? It is possible that sleep disturbance may be present at a specific time, e.g., at the early onset of psychopathology (a vulnerability window).

We would like to thank the reviewer for highlighting this interesting alternative interpretation.

Sadly, the battery of tests used in the HCP dataset does not consider symptomatology duration (whether related to psychopathology, affect or sleep complaints). We have revised the discussion to add this alternative interpretation to LC2.

Discussion – Page 9
"Another possible interpretation is that LC2 might reflect individuals who may lack insights into their sleep difficulties, particularly relative to other concerns that are more burdensome to them (i.e, difficulties functioning during daytime). Indeed, some individuals often fail to recognise the full impact of their sleep disturbances and attribute their daytime symptoms to external factors or normalise their tiredness(65). Nonetheless, whether this profile of sleep resilience or sleep misperception is a stable latent component, a cross-sectional observation of fluctuating symptoms that may develop into psychopathology-related sleep complaints or underlie objective sleep alterations, needs to be further tested.

It is mentioned that sleep disturbance in LC5 was associated with aggressive behavior (page 6, Figure 6). It would be informative to provide more information on the variable for aggression. Is it actually a behavioural task of aggression, or a self-report measure? Experimental research shows that sleep deprivation and restriction are associated with reduced aggressive behaviour on a laboratory task in men, a change that parallels reductions in testosterone following sleep loss.

The aggressive behaviour variable is extracted from the Achenbach Adult Self-Report questionnaire (Achenbach, T. M., & Rescorla, L. A. (2003), which is part of the mental health assessment (Table S1). The ASR is a 126-item self-report questionnaire for adults (aged 18–59) assessing aspects of adaptive functioning and problems, including scores for aggressive behaviour.

As the reviewer rightly points out, prior research would suggest a link between sleep duration and aggressive behaviour, though aggressive behaviour was not significant (i.e., not robust across participant resampling) in LC4. However, aggressive behaviour was significant in LC5, suggesting more of a link between aggressive behaviour and sleep fragmentation (i.e., multiple awakenings) rather than sleep duration per se.

Reviewer #3: Perrault et. al propose an original investigation of the multidimensionality of sleep and its links with the multidimension of socioeconomic and psychological factors on the one end (biopsychosocial factors - BPS) and brain function while awake one the other. They used data collected in >700 individuals as part of the Human connectome project with responses to a sleep questionnaire, many BPS variables and resting state fMRI recordings.
They decomposed the responses to a validated questionnaire in 7 dimensions that they related to >100 BPS features. They find that the first 5 (out of 7) latent components (LC) are "interpretable" are related to distinct set of BPS. They then report that these 5 latent components were significantly associated with distinct functional connectivity patterns.
While I salute the effort in proposing a novel view on sleep and the ambitious goal to make links between sleep BPS and brain function, I have several issues that leave me with a mixed impression of both positive and negative aspects. My main suggestion would be to remove a lot of the (unsure)

results to leave more space to the stronger findings that could be covered in more details.

Main issue

The main strength and also my main issue is the fact that LC1 explains 88% of the variance between sleep questionnaire dimensions and BPS. This is in itself remarkable and in my view the most interesting result. One would think that if sleep and BPS are multidimensional then their links should be diverse as well. According to the results, it is not the case and this deserves in my view a larger emphasis. The downside of the finding is that the other LCs only explain 4 to 1% of the variance. This raises questions about the relevance of these different LCs. Should the authors really focus on these LC? They should at least explain why it is interesting to focus on them and address the percentage of variance explained in their discussion.

We thank the reviewer for highlighting this important point. Indeed, LC1 encompasses a large part of the covariance between sleep and BPS measures. This association is driven by a general sleep factor and a general psychopathology (p) factor. Both the general sleep and psychopathology factors are driven by the high comorbidity among sleep and psychopathology measures. In turn, LC1 reflects a strong general (nonspecific) comorbidity between sleep and psychopathology shown in the data, supporting the view that sleep and psychopathology are intimately related.

However, the finding of LCs 2-7 being significant (and replicable across various control analyses) shows that other associations, driven by single sleep dimensions, also exist and are biologically relevant. The fact that they explain only a small part of the covariance might reflect that these are perhaps driven by a fraction of the participants. Alternatively, the associations captured by LCs 2-7 might also be present in all or most participants, but with less prominence.

We have updated the manuscript to be upfront about the fact that LC1 accounted for the vast majority of the covariance between sleep and biopsychosocial variables. Furthermore, we have restructured the discussion to be significantly more focused on LC1, and toned down the discussion around LCs 2-5:

<u>Results – Page 4</u>
"While LC1 accounted for a substantial amount of covariance between sleep and biopsychosocial measures, LCs 2-5 highlighted distinct covariance patterns that were driven by specific sleep dimensions, probably representative of only a fraction of the participants, or present in all or most participants but with less prominence."

<u>Discussion – Page 8-10</u>
We added more info on LC1 while reducing the paragraph on the other LCs

"We found that the first profile explained most of the variance and reflected general psychopathology (or p factor) associated with general poor sleep (LC1)."

"LC1 overwhelmingly explained 88% of the variance in a sample of healthy adults, highlighting the reciprocal relation between sleep and psychopathology and how sleep may be of utmost importance for both the prevention and treatment of mental disorders. LC1 reflected general psychopathology associated with overall poor sleep akin to insomnia complaints (i.e., difficulties falling asleep, maintaining sleep, sleep dissatisfaction, insufficient sleep). While we could not assess the presence of insomnia disorder based on clinical diagnostic criteria (54,55) or the chronicity of sleep complaints (i.e., PSQI captures sleep complaints only for the previous month)(39), there is a body of evidence of a reciprocal relationship between reports of poor sleep or insomnia complaints and psychopathology(56). Overall, poor sleep is not only a risk factor but also a co-morbid condition and transdiagnostic symptom for many mental disorders(57). When sleep is disrupted, it also contributes to the dysregulation of multiple neurobiological mechanisms related to emotional regulation and psychopathology(58). The strong co-morbidity between poor sleep and psychopathology is evident from a young age, as suggested by a recent study that used a similar data-driven approach (i.e., CCA) to assess associations between parent-reported sleep disturbances and a broad set of psychological, demographics and cognitive variables in a large sample of 9-10 year old children(59). The study reported a very similar latent component linking general poor sleep to general psychopathology, and that covariance pattern was replicated after a 2-year follow-up, suggesting the robustness of this association over time.

Although LC1 captured a large amount of covariance between sleep and biopsychosocial measures, LCs 2-5 showed covariance patterns that were characterised by specific sleep dimensions, which likely described associations seen in a fraction of the participants; alternatively, these associations might also be present in all or most participants, but with less prominence."

They should also explain what makes LC2 to 5 interpretable and L6-7 not interpretable while they explain about 1% of the variance (similar to LC5). Considering the supply. Fig. this feels unclear and rather subjective as I would see interpretation to both LCs.

LC6 and LC7 were considered less interpretable with regards to the sleep and literature by our sleep experts. In addition, the lack of either a single sleep dimension driving the component (as for LCs 2-5) or common directionality in sleep patterns complicated the interpretation. For example, LC6 is driven by difficulties falling asleep, but the associated biopsychosocial pattern is somewhat confusing, i.e., it is driven by showing avoidant personality problems, alcohol use (but not history of alcohol abuse), as well as low blood pressure, and short reaction time at the gambling task. Meanwhile, LC7 reflects low sleep efficiency and low sleep duration with short reaction time and good management of stress.

As suggested by Reviewer #1 and #3, we have now made changes to the manuscripts by acknowledging the predominance of LC1 and toning down all other covariance patterns (LC2-7). Nonetheless, we have added a description of LCs 6-7 in the Supplemental Results and have explained why we treat LC2-5 and LC67 differently in the Results.

Results – Page 4

"Out of the seven significant LCs that were derived, 5 LCs delineating multivariate relationships between sleep and biopsychosocial factors were supported by current sleep literature (Figure 1B; a description of LC6 and LC7 can be found in the Supplementary Results – Figure S1)."

Supplemental Results

"Latent Components 6 and 7 (LCs 6-7)

Compared to LCs 1-5, LC6 and LC7 were considered less interpretable and relevant with regards to established sleep literature. In addition, the lack of either a single driven sleep variable (like LC2-5) or common directionality in sleep patterns complicated the interpretation of the profiles. Nonetheless, the sleep-biopsychosocial covariance patterns are described below.

LC6 was characterised by longer sleep latency without sleep disturbance or sleep dissatisfaction. In terms of biopsychosocial variables, LC6 was driven by withdrawn and avoidant personality problems, short reaction time on a gambling task, low blood pressure, not being in a relationship and the use of alcohol (Figure S1A). Meanwhile, LC7 was driven by low sleep efficiency (i.e., long time spent in bed without sleeping) and short sleep duration as well as short reaction time on working memory and emotion processing cognitive tasks (Figure S1B)."

In any case whether they detail 5 or 7 LCs means that they then relate 5 or 7 LCs to functional brain activity. Each LC is related to distinct sets of brain connectivity pattern. Because of the multiplicity of the findings the reader is overwhelmed in results which are not discussed in detail. In the end it is unclear why the functional connectivity results- acquired during wakefulness- tell us something important about the sleeping brain, the BPS and the subjective sleep dimensions.
The issue is in my view also very relevant because the statistical approach used by the authors, Canonical Correlation analysis (CCA) can be unstable depending on whether a dimension reduction is carried out or not (depending therefore on multicolinearity of the variables (see e.g. Yang et al. 2021 HBM). While it is likely not going to drastically change the profile of LC1 given its prominence, it may change the other LCs. This raises further concerns about the relevance of LC2-7 and warrants demonstration of the stability of the CCA analyses.
All this pleads in my view in rewriting the paper only including LC1 and its brain correlates (at least in the main text). This is in my view the results one can be most sure of and they are novel and interesting (though I leave the editor chose whether it is interesting enough for their journal)

In addition to emphasising the discussion on LC1 and toning down LCs 2-5, we have computed a sensitivity analysis to test the reviewer's hypothesis on whether LCs other than LC1 are unstable, we have computed CCA after reducing the dimensionality of the biopsychosocial measures. We applied PCA on the biopsychosocial variables and re-computed a CCA between the 7 sleep variables and 67 PCA scores that explained 90% of the variance among the 118 biopsychosocial variables. Just like in the original CCA analysis, we found all 7 LCs to be significant ($ps < 0.001$). After matching the LCs between the two models using Hungarian algorithm, we compared the loadings from both models, and found strong correlations between the LC loadings from the original model and the PCA-CCA model, as shown in the following table (note that the sign of the CCA is arbitrary, e.g.,-0.9 and 0.9 are to be interpreted similarly):

	Correlation with original sleep loadings	*Correlation with original biopsychosocial loadings*
LC1	0.7924	0.9912
LC2	0.6589	0.7378
LC3	0.5957	0.5586
LC4	-0.8606	-0.8451
LC5	-0.7307	-0.7633
LC6	-0.9387	-0.8954
LC7	-0.8268	-0.6902

LC3 does seem less robust than the other LCs, but other LCs are not necessarily less robust than LC1 after dimensionality reduction was applied.

We now report this sensitivity analysis in the Methods and Results section, as well as in the Supplemental Results.

Methods – Page 15
"Sixth, to test the stability of our LCs, we re-computed the CCA after reducing the dimensionality of the biopsychosocial variables and using the principal components that explained 90% of the variance among the 118 biopsychosocial variables."

Results – Page 8 and Supplemental Results (Table S5)
"Second, we re-computed the CCA analysis after: (i) applying quantile normalization on sleep and biopsychosocial measures; (ii) excluding participants that had tested positive for any substance on the day of the MRI; (iii) excluding physical health measures (i.e., body mass index, hematocrit, blood pressure), (iv) excluding sociodemographic variables (i.e., employment status, household income, school status, relationship status) from the biopsychosocial matrix; (v) *after using PCA to reduce the dimensionality of the biopsychosocial variables;* and (vi) after considering only female (or male) participants in the CCA."

Furthermore, an important and novel part of our findings resides in the identified sleep-biopsychosocial profiles displaying unique patterns of brain network organisation, which is commonly measured via RSFC. Importantly, the associated RSFC patterns were not part of the CCA model but were computed post hoc in relation to averaged sleep/BPS scores. While RSFC was acquired during wake, FC measures provide important insights into the underlying functional brain network architecture, and suggest biological underpinnings to the covariance patterns between sleep and biopsychosocial measures. Interestingly, the differences between LC-RSFC profiles were mostly dependent on alterations to the segregation/integration ratio of the somatomotor and visual cortex, as well as differences in subcortical-cortical connectivity.

We discussed these further in the new Discussion- Page 9-10

"Interestingly, distinctions between LC1 and LC2 were also present in the neural signatures of RSFC, which may assist in the neurobiological interpretation of the profiles. Visually inspecting LC1 and LC2 suggested an underlying increase in subcortical-cortical connectivity when sleep disturbances are associated with psychopathology. This is in alignment with the known neurophysiology of the ascending arousal system and possibly implies the existence of some level of hyperarousal in these pathways that may contribute to disturbances in sleep(66). However, this speculation requires further targeted research to be confirmed."

"Interestingly, the strong RSFC patterns associated with LC4 showed a global increase in connectivity, alongside localised segregation of part of the somatomotor network. Similar patterns have been reported in neuroimaging studies of experimental acute total sleep deprivation(74,75) and were recently found to be associated with sleep duration in adolescents(16). This suggests that LC4 may reflect underlying sleep debt in the general population."

"Each LC presented a unique pattern of brain network organisation. Interestingly, alterations to the segregation/integration ratio of the somatomotor and visual cortex were common in most profiles. Highly interconnected to the whole brain, the somatomotor network is crucial for processing external stimuli and producing motor responses but is also functionally involved in bodily self-consciousness and interoception. Altered dysconnectivity patterns of the somatomotor network have been linked to variation in several domains, including general psychopathology(83,84), cognitive dysfunction related to sleep deprivation(74), as well as the total PSQI score(13,85). Overall, these findings suggest that alterations to RSFC in the somatomotor network are also involved in the relationships between sleep and biopsychosocial factors and highlight the importance of better understanding the role of this brain network in overall mental health and functioning."

Other important issues

- An issue that I would like to see addressed in the discussion is that CCA is inherently correlational, meaning that what composes an LC is driven by both sides of the correlation. The sleep questionnaire is therefore related to brain connectivity but also all the BPS associated to it in each LC. It is therefore at least plausible that the brain activity/connectivity recorded during wakefulness drives the BPS which are related to wakefulness in many instances which in turns is related to sleep dimensions.

The reviewer is correct in stating that the CCA is correlational and that an LC is driven by both sides of the equation, in this case, sleep and biopsychosocial measures. The connectivity measures were related to the CCA components *post-hoc*, thus, they will not have influenced the relationships between sleep and biopsychosocial measures. However, we acknowledge the reviewer's comment to review the RSFC associations in the context of biopsychosocial measures as well, and we included this in the discussion:

Discussion- Page 9-10

"Interestingly, the strong RSFC patterns associated with LC4 showed a global increase in connectivity, alongside localised segregation of part of the somatomotor network. Similar patterns have been reported in neuroimaging studies of experimental acute total sleep deprivation(74,75) and were recently found to be associated with sleep duration in adolescents(16). "

"Each LC presented a unique pattern of brain network organisation. Interestingly, alterations to the segregation/integration ratio of the somatomotor and visual cortex were common in most profiles. Highly interconnected to the whole brain, the somatomotor network is crucial for processing external stimuli and producing motor responses but is also functionally involved in bodily self-consciousness and interoception. Altered dysconnectivity patterns of the somatomotor network have been linked to variation in several domains, including general psychopathology(83,84), cognitive dysfunction related to sleep deprivation(74), as well as the total PSQI score(13,85). Overall, these findings suggest that alterations to RSFC in the somatomotor network are also involved in the relationships between sleep and biopsychosocial factors and highlight the importance of better understanding the role of this brain network in overall mental health and functioning."

We have also cited this as a potential limitation in the Discussion:

Discussion - Page 11

"The use of multivariate approaches provides insights into the multidimensional nature of sleep and/or biopsychosocial outcomes(15,21,30–32,59,88). *However, it is important to note that our findings, driven by CCA and RSFC correlations, do not inform on the directionality or causality of these effects."*

- I salute the inclusion of many control analyses which shows the will of the authors to back up their findings using enough information so that it is clear to the reader. Reporting that changing the processing using CompCor instead of GSR leads to r~0.75 is, however, not convincing as it means that only 50% of the variance is common among both methods. The situation is even worse for when using sleep and biopsychosocial canonical scores instead of averaged scores where r~0.5.

We thank the reviewer for raising this important point. The preprocessing of fMRI data is a critical and contentious aspect of neuroimaging research. However, there is significant debate over the optimal methods and their order of application, particularly the impact of global signal regression, and the best practices for motion correction and the handling of physiological noise. It is well understood that these choices can substantially affect results, potentially leading to different interpretations of the same data. For example, the differences in RSFC-motion correlations substantially differ between GSR + motion regression (r=0.058) and CompCor (r=0.17) [Ciric et al. 2017]. We have previously published such findings showing how global signal regression strengthens the association between resting-state functional connectivity and behaviour [Li et al, 2019]. Therefore, it is to be expected that differences may be large depending on the choice of preprocessing, but that is beyond the scope of this paper.

However, we understand the reviewer's point and have updated our reference to this in the Results and Discussion sections.

Results - Page 8
"We also assessed the robustness of our imaging results in several ways. First, we re-computed the GLM analysis using RSFC data that underwent CompCor(49) instead of GSR. The RSFC patterns were altered, although the patterns shared generally high correlations with the main analysis for most of the LCs (r=0.75, r=0.76, r=0.78, r=0.51, r=0.77 for LCs 1-5, respectively; Figure S3).

Discussion - Page 11
"Furthermore, whether or not participants fell asleep in the scanner did not impact our RSFC findings; however, the choice of preprocessing may have had repercussions on our results. The current state of neuroimaging suffers from a lack of consistency and agreement on preprocessing techniques, and these have been shown to alter relationships between RSFC and behaviour (93). While we acknowledge this limitation, a comprehensive investigation into the impact of preprocessing is beyond the scope of this paper."

References:

- Ciric R, Wolf DH, Power JD, Roalf DR, Baum GL, Ruparel K, Shinohara RT, Elliott MA, Eickhoff SB, Davatzikos C, Gur RC, Gur RE, Bassett DS, Satterthwaite TD. Benchmarking of participant-level confound regression strategies for the control of motion artifact in studies of functional connectivity. Neuroimage. 2017 Jul 1;154:174-187. doi: 10.1016/j.neuroimage.2017.03.020. Epub 2017 Mar 14. PMID: 28302591; PMCID: PMC5483393.
- Li J, Kong R, Liégeois R, Orban C, Tan Y, Sun N, Holmes AJ, Sabuncu MR, Ge T, Yeo BTT. Global signal regression strengthens association between resting-state functional connectivity and behavior. Neuroimage. 2019 Aug 1;196:126-141. doi: 10.1016/j.neuroimage.2019.04.016. Epub 2019 Apr 8. PMID: 30974241; PMCID: PMC6585462.

Also, I may have missed it but while the authors report that education level is related to at least 3 LCs (incl. LC1) they do not compute a control their analyses using education. Again, education may drive difference brain connectivity patterns and sleep dimensions.

We apologise if our manuscript lacked clarity about the way education level was controlled for in our analyses. We did investigate the effects of age, sex and education level in a sensitivity analysis in which we computed a CCA without regressing out these confounds, and found significant associations between education level and LC1, LC4 and LC5. However, in our main CCA analysis, we did correct for age, sex and education level. Therefore, the brain connectivity patterns are not likely to be driven by education. We have revised the Methods section to add details about the covariates that were adjusted for in the CCA.

Methods – Page 13

“Canonical Correlation Analysis (CCA)(102,103), a multivariate data-driven approach, was applied to the sleep and biopsychosocial measures, after regressing out the effects of age, sex, and education from both the sleep and biopsychosocial variables.”

- The main analyses include many subjects which were likely to have consumed psychoactive component prior to the fMRI acquisition. These were removed in a control analysis and it seems that it does not alter the findings. It would make much more sense to remove these individuals in the first place in a study investigation rs-fMRI.

We thank the reviewer for this remark. We chose to include individuals who had consumed psychoactive drugs on the day of the MRI acquisition, as we favoured a more naturalistic analysis. We were also interested in whether substance use impacts LC profiles and post-hoc RSFC analyses in a sample of the general healthy population. The lack of difference is interesting and worth mentioning, in our opinion. Our findings suggest that substance use in that context is not a confounding variable and thus allow for more naturalistic analyses in a general and diverse sample of the population. Nonetheless, we have cited this as a limitation in the Discussion:

<u>Discussion</u> - Page 11
“Finally, we chose to include participants who were likely to have consumed psychoactive drugs on the day of the fMRI acquisition, as we favoured a more naturalistic analysis; however, we showed that excluding these participants did not have any impact on our findings. Moreover, the RSFC patterns we found had been previously described in the sleep literature, suggesting that they are likely robust to these effects. “

- The fact that the sleep metrics, derived from a questionnaire, are inherently subjective should be further discussed as well. It is true that it makes the findings very practical/clinical (as mentioned by the authors) but does not tell us all about the underlying biology. Can the authors truly envisage an intervention/treatment based on their findings, shouldn't they need actimetry and/or electrophysiology to get to intervention targets?

We thank the reviewer for raising this important point. In the HCP dataset, sleep and most of the biopsychosocial measures are self-reported and thus dependent on the participant’s perception of their sleep and health. However, we argue that one’s perception of their sleep is clinically more meaningful than objective measures (e.g., polysomnography, actimetry). In the case of sleep for example, insomnia diagnosis (DSM-V, ICSD-3) is solely based on subjective reports of sleep complaints and does not require any objective markers. Thus, most RCT and experimental interventions will rather prioritise the use of self-reported measures (e.g., sleep diaries, insomnia severity index, PSQI) as primary outcomes. The use of actimetry and polysomnography allows for further understanding of sleep without systematically reflecting subjective sleep quality (i.e., sleep state misperception). However, we do agree that a similar study with objective sleep markers might reveal some interesting and clinically meaningful differences. While self-report measures (particularly the PSQI for sleep quality) have some limitations, this paper paves the way for future research to examine the LCs reported here using more objective measures of sleep.

We added this argument in the discussion as part of the study's limitations.

Discussion - Page 11

"A final important distinction to be addressed is that sleep and biopsychosocial outcomes were mostly self-reported through questionnaires. Both objectively recorded and subjectively perceived estimations provide different yet meaningful information that tends to positively correlate(95). However, it has been shown that when compared to objective estimates (i.e., polysomnography and/or actigraphy recordings), individuals with sleep complaints (i.e., chronic insomnia, obstructive sleep apnea) tend to subjectively misperceive their sleep (i.e., duration, sleep latency)(27,28,96,97). The degree of discrepancy between objective and subjective measures (i.e., sleep state misperception) has been correlated with worse sleep quality(98,99) as well as compromised reports of daytime functioning(26). While objective measurements might have exposed divergent associations between sleep and biopsychosocial factors, the profiles reported here arguably support greater clinical validity, as subjective complaints are often what drives an individual to seek out healthcare. It also paves the way for future research to examine the LCs reported here using more objective measures of sleep."

- The manuscript makes no mention about time-of-day or prior sleep-wake history of the fMRI recording. Whether acquisitions were completed at the same time of day for each subject is not mentioned. One can imagine many reasons why BPS / Sleep quality could be related to chronic sleep restriction or to fMRI acquisitions being completed at a particular time of day. This could induce a systematic bias in the analyses which should ideally be controlled for and at minimum discussed.

We thank the reviewer for this remark. We did take into account the sleep-wake history of the fMRI recording in a control analysis and removed participants who likely fell asleep in the scanner from the analysis, which did not alter the results (see Control analyses, Table S5, and Figures S3 and S4). As for time of day, fMRI was acquired over two sessions across two separate days, and the time of day differed across the two sessions. In our analyses, the RSFC was averaged across runs and sessions, therefore, we are not able to distinguish the time-of-day effect in our findings. However, the impact of time of day on RSFC has previously been described (Orban et al., 2020), and it might have impacted the RSFC patterns related to the LCs. We have now added this as a limitation in the Discussion:

Discussion - Page 11

"Furthermore, whether or not participants fell asleep in the scanner did not impact our RSFC findings; however, the choice of preprocessing may have had repercussions on our results. The current state of neuroimaging suffers from a lack of consistency and agreement on preprocessing techniques, and these have been shown to alter relationships between RSFC and behaviour (93). While we acknowledge this limitation, a comprehensive investigation into the impact of preprocessing is beyond the scope of this paper. In addition, the time-of-day of the 2 sessions of

fMRI acquisition, which has been shown to also affect relationships between RSFC and behaviour(94), could have also impacted our RSFC findings"

Reference: Orban C, Kong R, Li J, Chee MWL, Yeo BTT. Time of day is associated with paradoxical reductions in global signal fluctuation and functional connectivity. PLOS Biol. 2020 Feb 18;18(2):e3000602.

Minor issues

- Introduction, second paragraph, 4th line: it is awkward to define sleep in terms of alertness. Do the authors mean daytime functioning?

We thank the reviewer for the chance to clarify this point. Quantity, quality, regularity, timing, and alertness have been defined as being key dimensions of sleep that have been consistently associated with health outcomes (Buysse, 2014 – Ref 9). Alertness refers to the ability to maintain attentive wakefulness. The combination of these 5 dimensions can affect our daytime functioning and health. We have added the Buysse 2014 reference at the end of this sentence for more clarity.

<u>Introduction</u>- Page 3
"Sleep can be defined by its quantity (i.e., sleep duration) and quality (i.e., satisfaction, efficiency), as well as in terms of regularity, timing, and alertness (9)."

- Figure 2 legend for panel (C) does not match with the text on page 13 under RSFC analysis where Fig2C is for uncorrected and 2D is for FDR-corrected network-wise beta coeff.

We thank the reviewer for spotting this error and have corrected the text accordingly.

<u>Methods</u> – Page 14
"For a complete view of the component-specific RSFC signatures, we plotted both the uncorrected region-wise GLM beta coefficients (e.g., Figure *2B)* and FDR-corrected network-wise GLM beta coefficients (e.g., Figure *2C)."*

- Page 10, no reference is provided for "the U-shaped relationship of sleep duration with biopsychosocial measures. »

Thank you for identifying these missing references. We added the reference (Knutson et al., 2006; Liu et al., 2020; Li et al., 2022) to the sentence and the following section on the U-shaped association between sleep duration and health.

<u>Discussion</u> – Page 11
*"*Future work is needed to extend these findings and further explore the multidimensional nature of sleep health, for instance, taking into consideration the U-shaped relationship between sleep duration and biopsychosocial measures*(63,89,90*). Given the design of the PSQI, only short sleep

duration (<5-6h) was considered as a sleep difficulty, neglecting the potential consequences of long sleep duration (>9h). Long sleep duration is commonly observed in hypersomnia disorders and psychopathology (e.g., schizophrenia, depression)(6,91), as well as being associated with increased risk of cardiovascular heart disease and mortality(7,92,93), *depression and cognitive decline(6,22,63,90). This U-shape relationship, whereby both short and long sleep durations are associated with negative impact on health and cognition as well as increasing markers of cerebrovascular burden (e.g., white matter hyper-intensities)(63,89), may provide a window to identify mechanisms that underlie the interplay between sleep and biopsychosocial factors.*

- The authors should consider discussing other multivariate papers such as Djonlagic et al. 2021 NHB which related objective measures of sleep to cognition and aging, which is not identical but somewhat similar to their study.

We thank the reviewer for suggesting this reference. We added this reference, as well as other sleep papers that used multivariate data-driven approaches, to the discussion.

<u>Discussion</u> – Page 10
"While unidimensional association studies are informative, our findings reinforce the notion that sleep health is multidimensional and distinct measures of sleep quantity or quality should be considered together when investigating their influence on biopsychosocial aspects of health, cognition, and lifestyle. The use of multivariate approaches provides insights into the multidimensional nature of sleep and/or biopsychosocial outcomes(15,21,30–32,59,88)."

- Page 12, the authors should provide details about the missing data and data removed. At the moment we know what was done but not how much data was missing / removed.

We thank the reviewer for the chance to clarify this point. The CCA requires complete data from all participants. As such, participants with any missing data on one or more measures of interest were excluded (N = 403) from the analyses. 33 participants with visual impairment that might have impacted their task performance in the scanner were also removed. We state this in the Methods section:

<u>Methods</u> - Page 12
"Of the 1,206 total subjects available from the HCP S1200 release, we excluded 403 participants with missing/incomplete data on one or more measures of interest, and 33 participants with visual impairment that might have impacted their task performance in the scanner."
