# Supplementary material for: Identification of five sleep-biopsychosocial profiles with specific neural signatures linking sleep variability with health, cognition, and lifestyle factors
Source: PLoS Biol. 2025 Oct 7;23(10):e3003399. doi: 10.1371/journal.pbio.3003399 (PMC12503254; doi:10.1371/journal.pbio.3003399)
Supplement: Attachment — Submitted filename: Response_to_reviewers.pdf [file pbio.3003399.s006.pdf]

We thank the reviewers for their time in reviewing our work and providing feedback. Please find our responses written in blue. Changes in the manuscript are provided in *blue and italics*.
Find the response to editor in the new cover letter.

Reviewer #1:

1. "LC1 overwhelmingly explained 88% of the variance in a sample ..." is incorrect. Authors can estimate the explained variance via calculating out-of-sample r between the two sides (which is maximum going to be .69 based on the Figure 1, thus estimated R2 will be maximum 48%). The 88% is the proportion of covariance between sleep scores and BPS scores captures by the LC1 (not variance in the sample).

The reviewer is correct in pointing that 88% is the amount of covariance, not variance, explained by LC1. We have changed the manuscript accordingly:

[Page 8] "We found that the first profile explained most of the covariance and reflected general psychopathology (or p factor) associated with general poor sleep (LC1)."

[Page 9] "LC1 overwhelmingly explained 88% of the covariance between sleep and biopsychosocial scores in a sample of healthy adults, highlighting the reciprocal relation between sleep and psychopathology and how sleep may be of utmost importance for both the prevention and treatment of mental disorders."

2. I would put a bigger caveat on LC3-5 as they are not only smaller in capturing covariance, but also seem to be not stable enough to cross-validate.

We agree with the reviewer and have added this caveat at the beginning of the Results section, before describing the LCs:

[Page 5] "While LC1 and LC2 were defined by general patterns of sleep (either general poor sleep or sleep resilience), LCs 3-5 reflected more specific sub-components of the PSQI, all associated with specific patterns of biopsychosocial factors; they also showed to be less robust and generalizable than LCs 1-2, as they did not survive cross-validation in our control analyses."

Reviewer #3:

The authors responded satisfactorily to most of my comments.
I still regret the limited discussion of the actual brain regions and/or networks in relation to each LC, and particularly to LC1. A lot of discussion is about the what the different LCs

represent in terms of BPS but much less what the brain region associated to the BPS/LCs tell us about sleep.

What does it tell us about sleep that the LC1 is associated with a negative connectivity between the TPN and DAN/SMN when the association is positive when considering TPB vs. DMN/CON. Likewise what do we learn about SubC or SAL connectivity (or absence of) considering LC1? Etc.
I realise that it is difficult to discuss all the associations uncovered given their large number. In my view, this makes the inclusion of the RSFC and of 5 (or 7) LC spectacular and/or technically very impressive rather than providing insights in the biology of sleep. I would recommend increasing the discussion about the brain areas/networks by selecting some of the most striking connectivity findings, according to the authors (and beyond the segregation/integration ratio of the somatomotor and visual cortex). Else the RFSC feels rather technical in my view.

We appreciate the reviewer's acknowledgement that describing every specific change in RSFC is difficult. However, we believe that these are still important findings that assist in the neurobiological interpretation of the LC profiles and are of interest to readers.

We have added further discussion of the associations with RSFC. We now explain these changes in the context of each LC throughout the discussion, and hope this provides further context for these findings and their importance in this study.

[Page 9]: "Visually inspecting LC1 and LC2 suggested an underlying increase in subcortical-cortical connectivity when sleep disturbances are associated with psychopathology. This is in alignment with the known neurophysiology of the ascending arousal system and possibly implies the existence of some level of hyperarousal in these pathways that may contribute to disturbances in sleep(66 The stronger RSFC between subcortical, somatomotor, and dorsal attention networks (DAN) in LC1, and the preserved thalamocortical and control network coupling in LC2, nevertheless suggest that these network patterns may reflect vulnerability and resilience pathways to sleep disturbances in psychopathology. However, this speculation requires further targeted research to be confirmed. The reduced connectivity between the temporoparietal network (TPN) and both the dorsal attention and somatomotor networks in LC1 may reflect a breakdown in the typical antagonism between internally and externally oriented brain systems(67), potentially facilitating maladaptive self-referential processing. This pattern aligns with prior work linking increased default mode network (DMN) connectivity to rumination in depression(68,69) and extends these findings by suggesting that such connectivity profiles may underlie transdiagnostic vulnerability to poor sleep quality. The absence of this pattern in LC2—despite similar levels of psychopathology—raises the possibility that disrupted TPN-DAN balance is not merely a

consequence of mental health symptoms but may reflect a mechanism contributing specifically to sleep disturbance..”

[Page 10]: “Interestingly, the strong RSFC patterns associated with LC4 showed a global increase in connectivity, alongside localised segregation of part of the somatomotor network. Similar patterns have been reported in neuroimaging studies of experimental acute total sleep deprivation (74,75) and were recently found to be associated with sleep duration in adolescents(16). These RSFC features are thought to reflect homeostatic mechanisms that regulate brain function and suggest that LC4 may index underlying sleep debt in the general population.”

[Page 10]: “LC3, defined by sleep-aid use and relative absence of daytime complaints, showed increased RSFC within the visual and default mode networks, and greater segregation in visual and limbic systems. This pattern, along with impaired visual memory and emotion recognition performance, may reflect sedation-related reductions in network integration(86) that disrupt perceptual and affective-cognitive processes, despite subjectively intact attention and daytime functioning.”
